# Multicellular contractility contributes to the emergence of mesothelioma nodules

**DOI:** 10.1038/s41598-020-76641-x

**Published:** 2020-11-18

**Authors:** Julia Tarnoki-Zach, Paul Stockhammer, Dona Greta Isai, Elod Mehes, Balint Szeder, Ildiko Kovacs, Edina Bugyik, Sandor Paku, Walter Berger, Sufi Mary Thomas, Zoltan Neufeld, Balazs Dome, Balazs Hegedus, Andras Czirok

**Affiliations:** 1 Department of Biological Physics, Eotvos University, Budapest, Hungary; 2 Department of Thoracic Surgery, Ruhrlandklinik, University Duisburg-Essen, Essen, Germany; 3 Division of Thoracic Surgery, Department of Surgery, Comprehensive Cancer Center, Medical University of Vienna, Vienna, Austria; 4 Department of Anatomy and Cell Biology, University of Kansas Medical Center, Kansas City, KS USA; 5 Institute of Enzymology, Research Centre for Natural Sciences, Budapest, Hungary; 6 National Koranyi Institute of Pulmonology, Budapest, Hungary; 7 First Department of Pathology and Experimental Cancer Research, Semmelweis University, Budapest, Hungary; 8 Department of Medicine, Institute of Cancer Research and Comprehensive Cancer Center, Medical University of Vienna, Vienna, Austria; 9 Department of Otolaryngology, University of Kansas Medical Center, Kansas City, KS USA; 10 School of Mathematics and Physics, University of Queensland, Brisbane, Australia; 11 Department of Thoracic Surgery, Semmelweis University and National Institute of Oncology, Budapest, Hungary; 12 Division of Molecular and Gender Imaging, Department of Biomedical Imaging and Image-guided Therapy, Medical University of Vienna, Vienna, Austria

**Keywords:** Biophysics, Cancer, Cell biology

## Abstract

Malignant pleural mesothelioma (MPM) has an overall poor prognosis and unsatisfactory treatment options. MPM nodules, protruding into the pleural cavity may have growth and spreading dynamics distinct that of other solid tumors. We demonstrate that multicellular aggregates can develop spontaneously in the majority of tested MPM cell lines when cultured at high cell density. Surprisingly, the nodule-like aggregates do not arise by excessive local cell proliferation, but by myosin II-driven cell contractility. Prominent actin cables, spanning several cells, are abundant both in cultured aggregates and in MPM surgical specimens. We propose a computational model for in vitro MPM nodule development. Such a self-tensioned Maxwell fluid exhibits a pattern-forming instability that was studied by analytical tools and computer simulations. Altogether, our findings may underline a rational for targeting the actomyosin system in MPM.

## Introduction

Cellular contractility is reported to facilitate spreading of malignant cells^1–4^. Here, we focus on the role of cell contractility in malignant pleural mesothelioma (MPM), a tumor arising from mesothelial cells that line the pleural cavity. This highly aggressive disease is characterized by rapid local recurrence and resistance to therapy. Accordingly, MPM has a poor prognosis with the majority of patients succumbing to disease. A characteristic feature of MPM is nodular pleural thickening, caused by the formation of multiple, macroscopic tumor nodules on the pleural surface. Unlike solid tumors forming within a tissue, MPM nodules, protruding into a cavity may have certain growth and spreading dynamics.

In malignant diseases, emerging evidence suggests mechanosensing and contractility as major drivers for tumor progression and dissemination^5^. In fact, during tumor progression, cancer cells frequently develop a more contractile phenotype in order to enable cancer cell invasion and metastasis, one of the hallmarks of cancer^6,7^. Accordingly, regulators of actomyosin contractility and Rho-ROCK signaling in cancer cells have become the main target of drugs that target cell motility (“migrastatics”) and showed promising effects both in vitro and in vivo in a variety of cancer types including lung, breast and prostate cancer^5,6^. Changes in the cytoskeletal intermediate filament composition—a pivotal factor in cell morphology and stiffness—also provide important diagnostic information. Accordingly, the expression of vimentin and various cytokeratins are diagnostic markers in the histopathology of pleural mesothelioma^8^. However, the clinical relevance of mechanosensing genes that are directly responsible for the conversion of cytoskeletal stress to transcriptional regulation—including SUN1/2, nesprin and plectin—has not yet been studied in mesothelioma^9^.

Cell contractility-induced forces exerting on the cell’s microenvironment represent an important mechanism of multicellular patterning. Epithelia are particularly well known to utilize cell contractility for morphogenesis. For example, an anisotropic contractile activity gives rise to cell intercalation, when cell adjacency changes in such a way that the whole tissue elongates in one direction while it narrows along the perpendicular direction^10,11^. Similarly, contractility-driven constriction of the free (apical) surface of the epithelium can give rise to bending or budding within an epithelial sheet^12^. Mesenchymal cells can also contract the surrounding extracellular matrix (ECM): if a cell aggregate is placed on the surface of a collagen gel, cell traction reorganizes the collagen and creates bundles of ECM that radiate away from the aggregate^13,14^. This observation has led to the development of the mechano-chemical theory of pattern formation^15,16^ according to which cells exert traction forces on an underlying deformable substrate and the resulting strain transports (convects) both cells and the ECM. Furthermore, strain-oriented ECM filaments can guide cell motility, as cells are more likely to move parallel with the orientation of the ECM^17–19^. This mechanism was suggested to guide vascular patterning, and endothelial cells were reported to be able to detect and respond to substrate strains created by the traction stresses of neighboring cells^20^.

In this study, we demonstrate that several MPM cell lines can form nodule-like aggregates in vitro when cultured at high cell density. Live imaging in the presence and absence of myosin II inhibitors reveal that cell contractility is the primary driver for nodule formation in vitro. The abundance of f-actin and myosin-containing stress cables in MPM surgical specimen as well as in MPM tumors grown in mice also indicate the presence of contractility in vivo. We propose a quantitative model to explain nodule formation within a contractile cell sheet. With stochastic simulations we examine the effects of key model parameters, specifically, the stability of cell-cell and cell-substrate adhesions, and the magnitude of cell-exerted contractile forces. Taken together, these studies may point to an important role of cell contractility in the pathophysiology of MPM.

## Results

### MPM nodule formation

**Figure 1:**
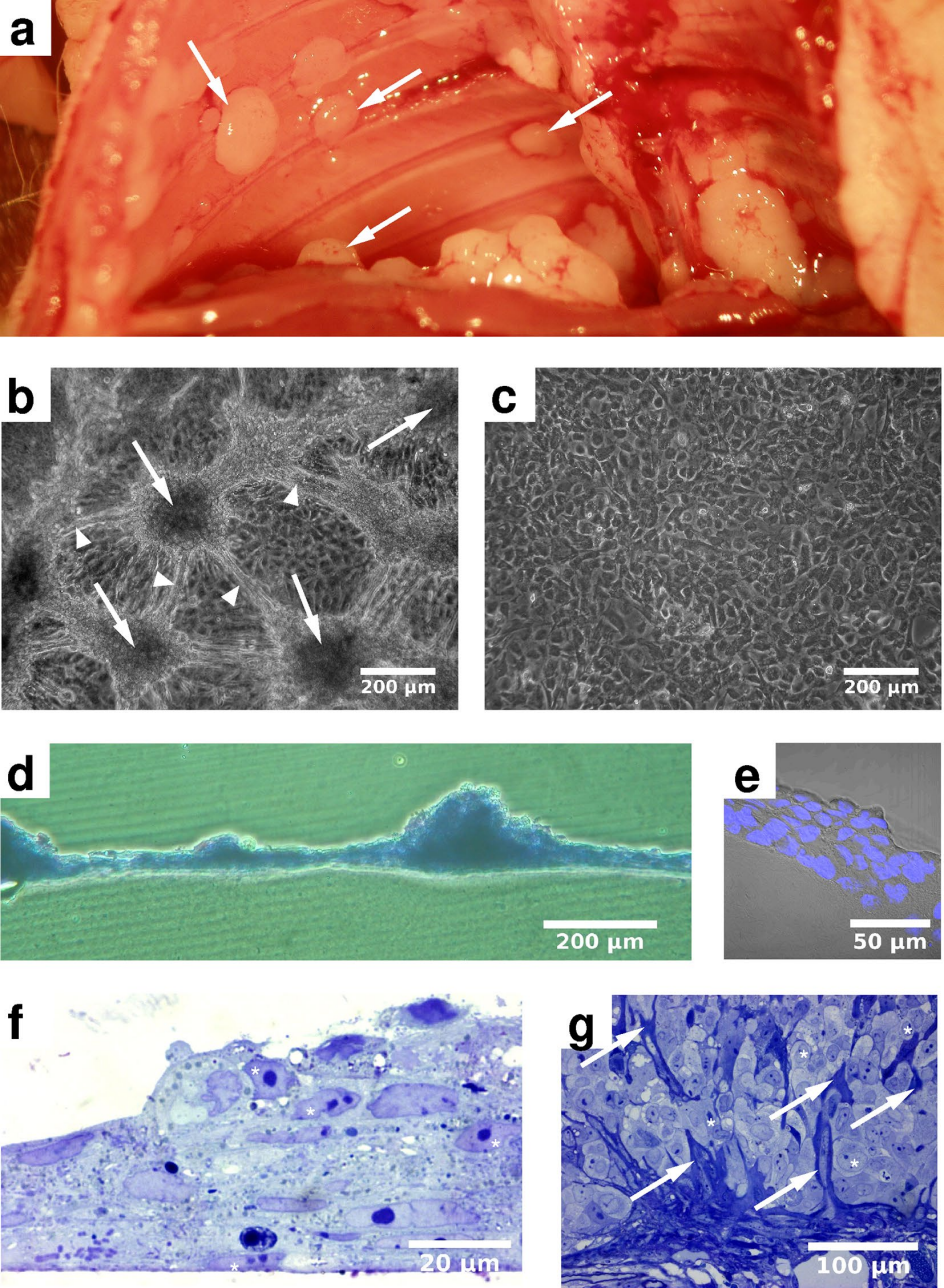
MPM cells form characteristic nodules both in vivo and in vitro. (**a**) SPC111 mesothelioma cells transplanted into a SCID (severe combined immunodeficiency) mouse ($\hbox {n}=10$) form nodules (arrows) within 21 days. (**b**) After a week in culture, SPC111 cells form nodules (arrows), interconnected by multicellular strands (arrowheads). Similar behavior was observed in $\hbox {n}=54$ independent experiments. (**c**) In contrast, human non-malignant primary mesothelial cells remain in a monolayer configuration up to 17 days in culture ($\hbox {n}=2$). (**d**, **e**) Physical, $100 \,\mu \hbox {m}$ thick sections of SPC111 nodules, formed on the surface of a collagen-I gel, harvested at culture day 11. (**d**) A low magnification image depicts $\sim 200 \mu \hbox {m}$ size nodules, interconnected by a thin, confluent layer of cells (toluidine blue-staining). (**e**) Higher magnification image of a nodule depicts DAPI (4,6-diamidino-2-phenylindole) stained nuclei in multiple layers. (**f**, **g**) Semithin sections ($0.5 \,\mu \hbox {m}$) of SPC111 nodules prepared from day 7 cultures (**f**), and from a SCID mouse xenograft 21 days after tumor inoculation (**g**). Both in vitro and in vivo samples contain prominent pleiomorphic nuclei (asterisks). The in vivo MPM nodule is rich in ECM (arrows).

To study the collective behavior of MPM cells, we performed long term live-cell imaging of confluent cultures. In such long term cultures around half of the cell lines established from MPM patients form macroscopic nodules (Supplementary Table S1), similar to nodules appearing on the pleural surfaces of mesothelioma patients and in orthotopic models of certain mesothelioma cell lines (Fig. 1a). These spontaneous multicellular aggregates can be a millimeter wide and $50\hbox {-}150 \,\mu \hbox {m}$ thick (Fig. 1b). Physical cross-sections (Fig. 1d–e) of the nodules reveal densely packed cellular clusters containing prominent, pleiomorphic nuclei. In vitro formation of similar nodules is less common (20%) in tumor cell lines of epithelial origin (Supplementary Table S1), however a few of those also formed nodules in vitro. Nodule formation was less common in non-malignant cultures: For example, HaCaT keratinocyte cells or the MDCK kidney tubule epithelial cells maintain a monolayer even at high densities^21^. Importantly, under similar culture conditions and duration, human non-malignant primary mesothelial cells also maintain a contact-inhibited monolayer (Fig. 1c). In a recent study we described the major molecular alterations for our MPM cell line panel^22^. Among the MPM tumor cell lines, we did not identify significant associations between tumor nodule formation and histological subtype or major molecular alterations in MPM including BAP1, TP53, NF2 or TERT promoter mutations (Supplementary Fig. S1 and Table S2).

**Figure 2:**
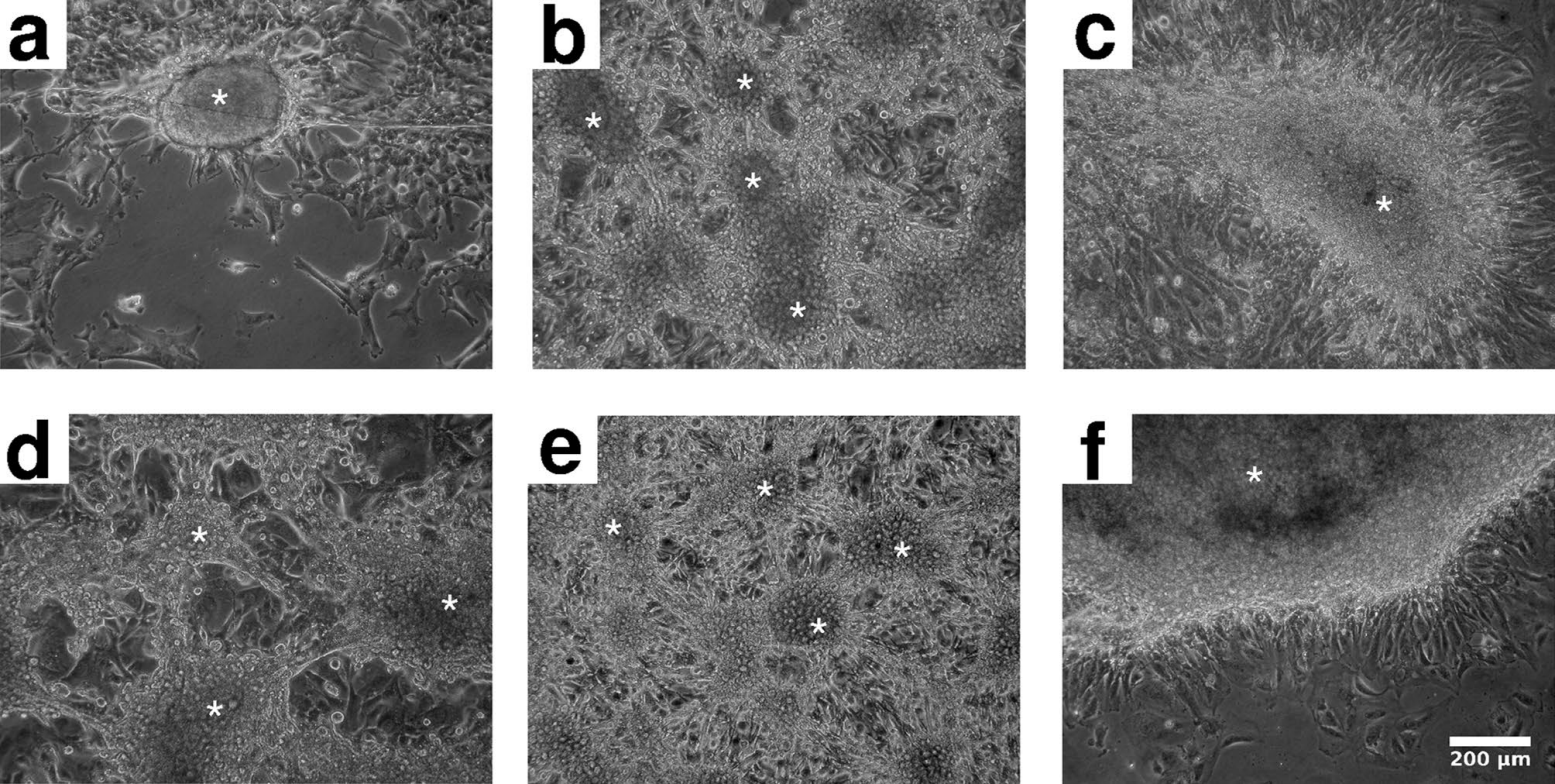
Nodule morphologies, formed spontaneously by human MPM cell lines in culture. Panels depict cultures of Meso62 (**a**), P31cis (**b**), Meso53 (**c**), VMC20 (**d**), VMC48 (**e**) and Meso80 (**f**) MPM tumor cells after one (**b**, **d**, **e**) or two (**a**, **c**, **f**) weeks in culture. Asterisks mark nodules. Nodule formation was present in 4 out of 4 (Meso62), 11 out of 11 (P31cis), 3 out of 4 (Meso53), 16 out of 16 for VMC20, 7 out of 7 for VMC48 and 6 out of 6 (Meso80) independent experiments.

The SPC111 cell line exhibits a monolayer of epithelioid cells between the nodules and their interconnecting multicellular strands (Fig. 1b,d). The two cell phenotypes (epithelioid vs nodule-forming) can be interconverted as cells reseeded from a nodule give rise to both populations. Such a dual phenotype of MPM tumor cells, however, is not always present: the Meso62, Meso53 and Meso80 cell lines form nodules without maintaining a continuous epithelioid layer (Fig. 2).

Surprisingly, nodules develop even in the absence of cell proliferation. Hydroxyurea (HU), an inhibitor of deoxyribonucleotide production, can effectively arrest the cell cycle and block cell divisions (Supplementary Fig. S2). In particular, HU-treated SPC111 tumor cells are vital and able to engage in motility—yet growth measured by the total protein content can be arrested in a dose-dependent manner (Supplementary Fig. S2). Since nodules readily form in cultures of HU-treated SPC111 cells (Supplementary Fig. S2), in vitro nodules are not clusters of highly proliferative cells.

As live imaging studies indicate, in vitro nodule formation involves substantial cell movement (Supplementary Fig. S3 – S4, Supplementary Movies S1 and S2). After an initial lag phase, both SPC111 and VMC20 tumor cells aggregate at the sites of future nodules. Kymograms of the process indicate a gradual convergence which is approximately linear in time (Supplementary Fig. S3 and S4 for SPC111 and VMC20 cultures, respectively). To quantitatively characterize the aggregation process, we used two measures. One is the overall convergence within a field of view, as defined in^23^. This quantity evaluates the velocity field at each frame, and thus provides a time-resolved measure of contractility (Supplementary Fig. S3–S5). The time-averaged contractility is $9.5 \pm 0.2$%/h ($\hbox {n}=13$), $14.8 \pm 0.4$ %/h ($\hbox {n}=8$) and $17.6 \pm 0.3$ %/h ($\hbox {n}=5$) for the most contractile SPC111, p31 and VMC20 cell lines, respectively. To characterize the cumulative effect of agregation dynamics, we also analyzed the distribution of virtual tracer particles that move with the local image details as described in^24^ (Supplementary Movie S3). For each time point *t*, the non-uniform spatial distribution of the tracer particles is determined by laying a uniform square grid over the field of view, and calculating the number of tracer particles in each lattice cell. The standard deviation of the lattice occupancy values, *S* is then also characterizes the aggregation process (Supplementary Fig. S3–S5 for SPC111 and VMC20 cultures, respectively). The linear increase in *S*(*t*) is consistent with the gradual convergence seen in Supplementary Figs. S3 and S4.

### Nodule formation is driven by cell contractility

**Figure 3:**
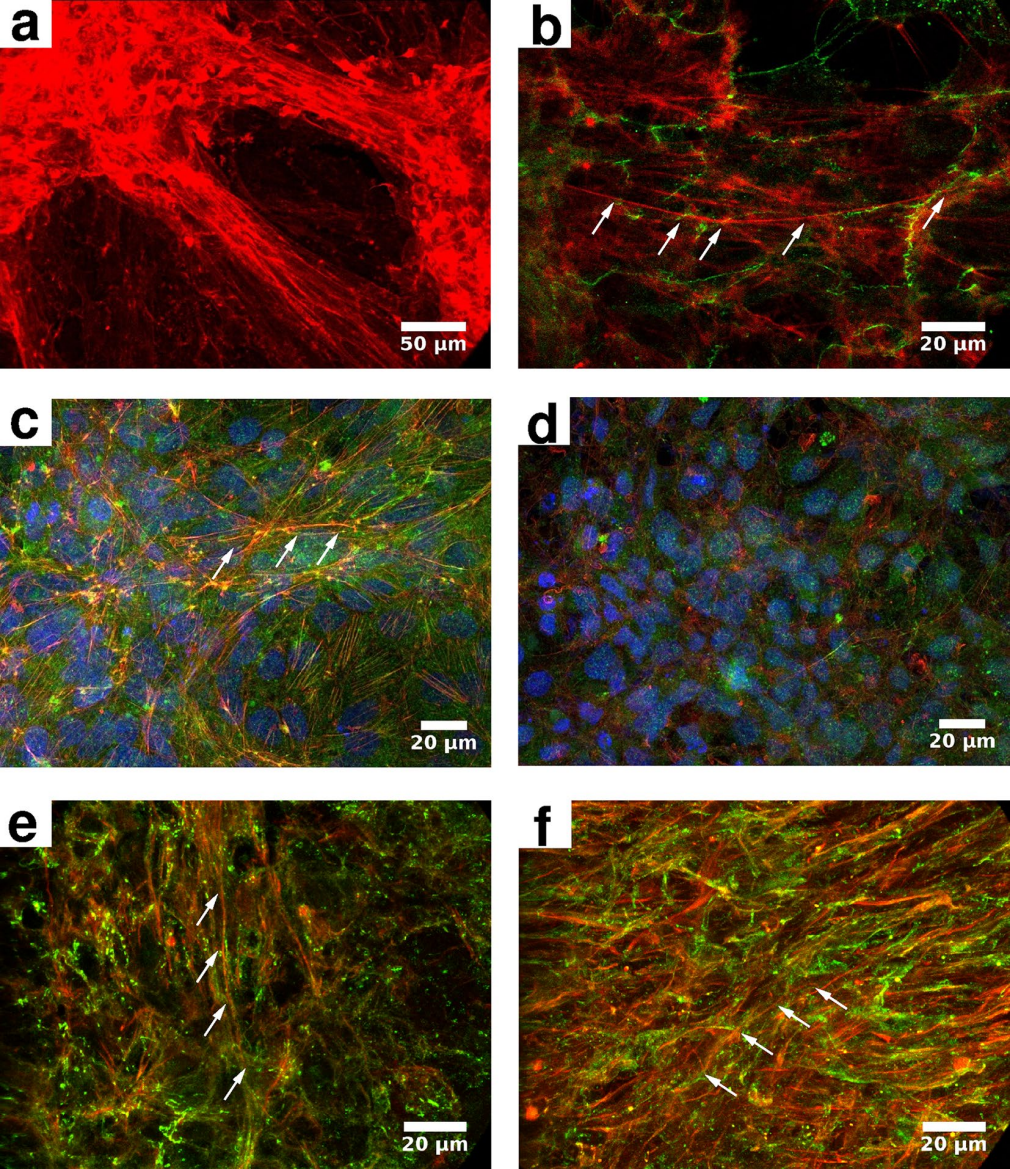
MPM nodules are rich in stress filaments and are mechanically integrated. Actin filaments are visualized by confocal microscopy using phalloidin (red). (**a**–**d**) SPC111 nodules collected from cell culture. A confocal z-projection (**a**, 40x objective) indicates that f-actin (red), organized into stress filaments (arrows), is profound within the nodules and their interconnecting strands, but not within the monolayer of SPC111 cells (compare with Figure 1c). (**b**) Higher magnification reveal that stress cables span multiple cells (arrows) and align across cell membranes (100x objective, single confocal section). Cell membranes are visualized by beta-catenin antibodies (green). (**c**) Actin filaments (red, arrows) co-localize with phospho-Myosin II (green, 40x, confocal z-projection). Nuclei are visualized by DAPI (blue) in (**c**, **d**). (**d**) When myosin II activity is inhibited by $100 \,\mu \hbox {M}$ Y27632, stress cables are largely absent (40x objective, confocal z projection). (**e**, **f**) Multicellular stress filaments (red, arrows) are also present in xenograft tumors from SCID mice (**e**) as well as in human surgical specimen (**f**, green: beta-catenin, 100x objective, confocal z-projection).

**Figure 4:**
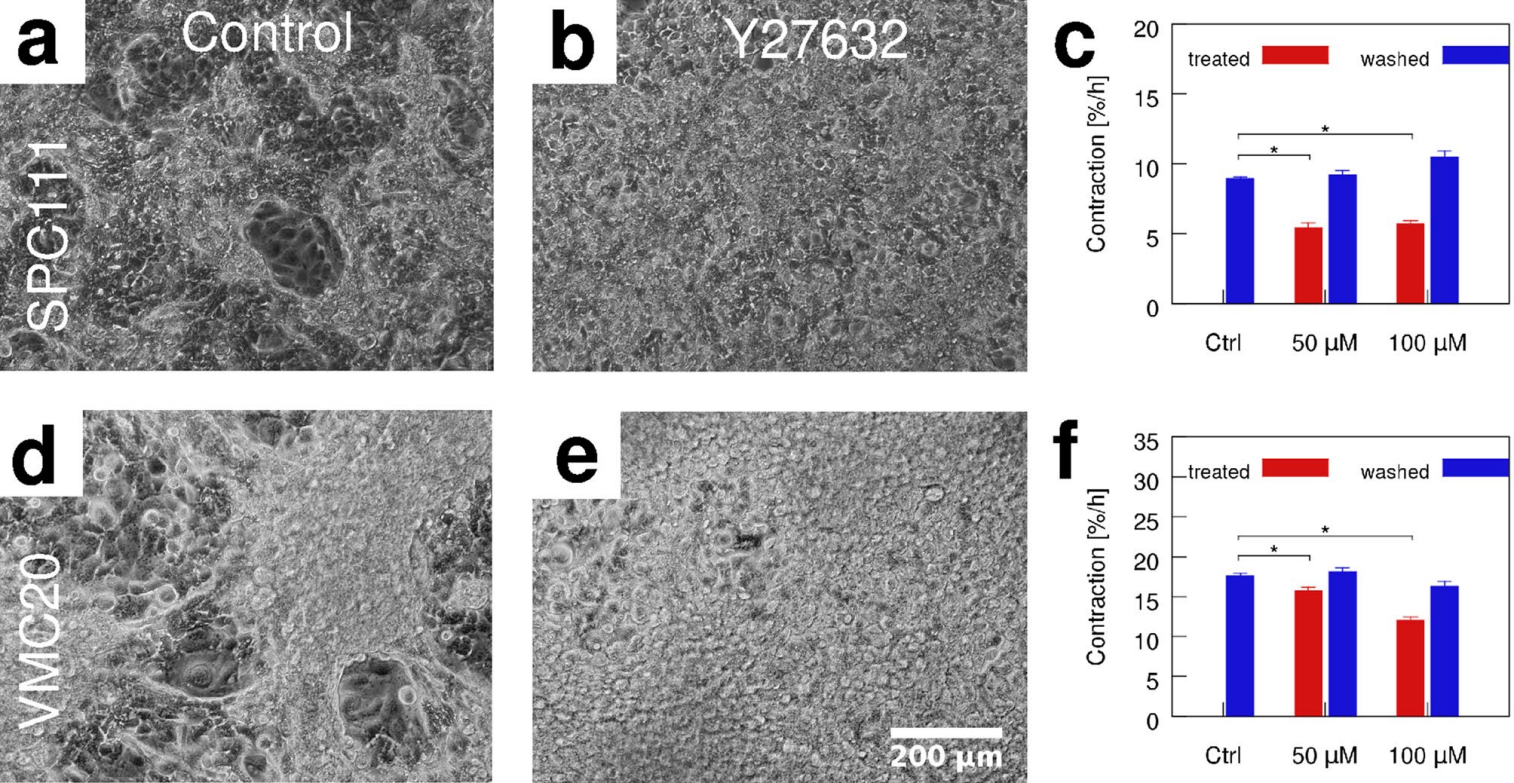
Nodule formation in cultures of SPC111 (top) and VMC20 (bottom) MPM cell lines, in the presence and absence of normal Myosin II activity. In untreated control cultures (**a**, **d**), nodules develop within 7 days. Such aggregates are absent in both SPC111 (**b**) and VMC20 (**e**) cultures treated with 50 or $100 \,\mu \hbox {M}$ ROCK inhibitor Y27632. Average nodule contractility was quantified in untreated cultures, during treatment by 50 and 100 uM Y27632 Rho kinase inhibitor and after the washout of the drug, both in SPC111 (c, $\hbox {n}=4$, $p=5\cdot 10^{-8}, 10^{-12}$ ) and VMC20 (f, $\hbox {n}=3$, $p=7\cdot 10^{-4},3\cdot 10^{-10}$) MPM cultures. Statistical significance was established by 2-tailed, heteroscedastic t-tests (asterisks). Error bars represent standard error of the mean.

**Figure 5:**
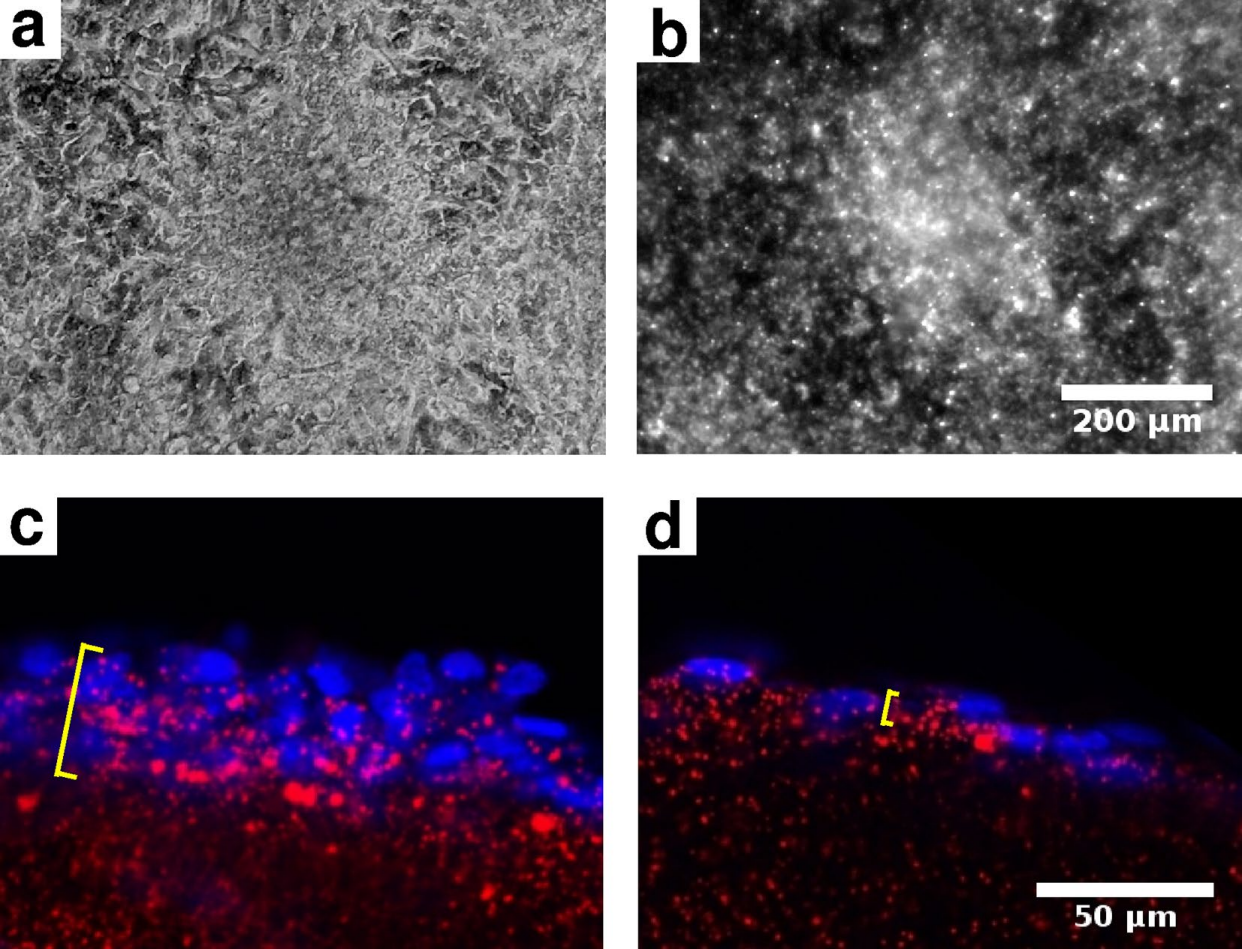
Contractile mesothelioma nodules incorporate elements of the tissue microenvironment in vitro. VMC20 cells were grown on the surface of fluorescent microbead-containing collagen-I gel substrate. A nodule and its microenvironment are shown after 5 days in culture, visualized by phase-contrast (**a**) and epifluorescence (**b**) microscopy, respectively. Confocal images of $100 \,\mu \hbox {m}$ thick physical sections reveal accumulation of microbeads (red) within the nodule (**c**, bracket). In contrast, the surface width of the bead-containing collagen gel is less than $10 \,\mu \hbox {m}$ in similar physical sections of VMC20 monolayers (**d**, bracket). Cell nuclei are labeled with Hoechst 33342 (blue).

Visualization of actin filaments with fluorescent phalloidin (Fig. 3) reveals that nodules are rich in stress cables. Prominent stress cables are organized into parallel bundles which mechanically connect adjacent nodules (Fig. 3a). Higher resolution confocal images indicate that stress filaments reach across cell bodies and form structures that are continuous—at the resolution of optical microscopy—across cell membranes (Fig. 3b,c). Multicellular stress cables are also abundant in human MPM surgical specimens (Fig. 3e) and in tumors of immunocompromised mice initiated by human MPM xenografts (Fig. 3f).

Both the observed cell movements and the presence of prominent stress cables within the aggregates suggest that acto-myosin contractility is an important mechanism to drive MPM cells into nodules. To test this hypothesis, we administered drugs that interfere with normal myosin II activity. Blebbistatin stabilizes type II myosins in the low-affinity actin binding conformation, hence it is a potent allosteric inhibitor of acto-myosin contractility^25^. The compound Y27632 is a specific inhibitor of Rho kinase (ROCK)^26^, which is a prominent myosin activator.

Both Y27632 and blebbistatin substantially reduce or completely abrogate the formation of intracellular stress cables (Fig. 3d) as well as the formation of multicellular nodules when cells were exposed to myosin inhibitors from the time of plating (Fig. 4, Supplementary Movies S1–S2). Previously formed nodules reversibly flatten and expand when exposed to either inhibitor (Supplementary Fig. S3–S5). Three days after removal of the inhibitor, nodule morphologies resemble those observed in untreated control cultures (Supplementary Fig. S3–S5). Contractile activity was quantitatively characterized by the divergence of the velocity field, a technique used previously to study cardiomyocyte phenotype in vitro^23,27^. Administration of Y27632 reduces contractility by 50% and 40% in SPC111 (Fig. 4c) and VMC20 (Fig. 4f) cultures, respectively.

The contractility of MPM nodules may also provide a previously unknown mechanism to incorporate elements of the normal tissue environment into the tumor. To test this hypothesis, VMC20 cells were cultured on the surface of Collagen-I hydrogels that included fluorescent marker beads (Fig. 5). MPM nodules that developed under such culture conditions incorporated beads in the bulk of the aggregate (Fig. 5b,c, Supplementary Movie S5). Thus, the ECM-rich compartments seen in SPC111 xenograft tumors (Fig. 1g) could arise through contractility-driven internalization of the surrounding stroma.

### Cell-resolved simulations of a contractile monolayer

**Figure 6:**
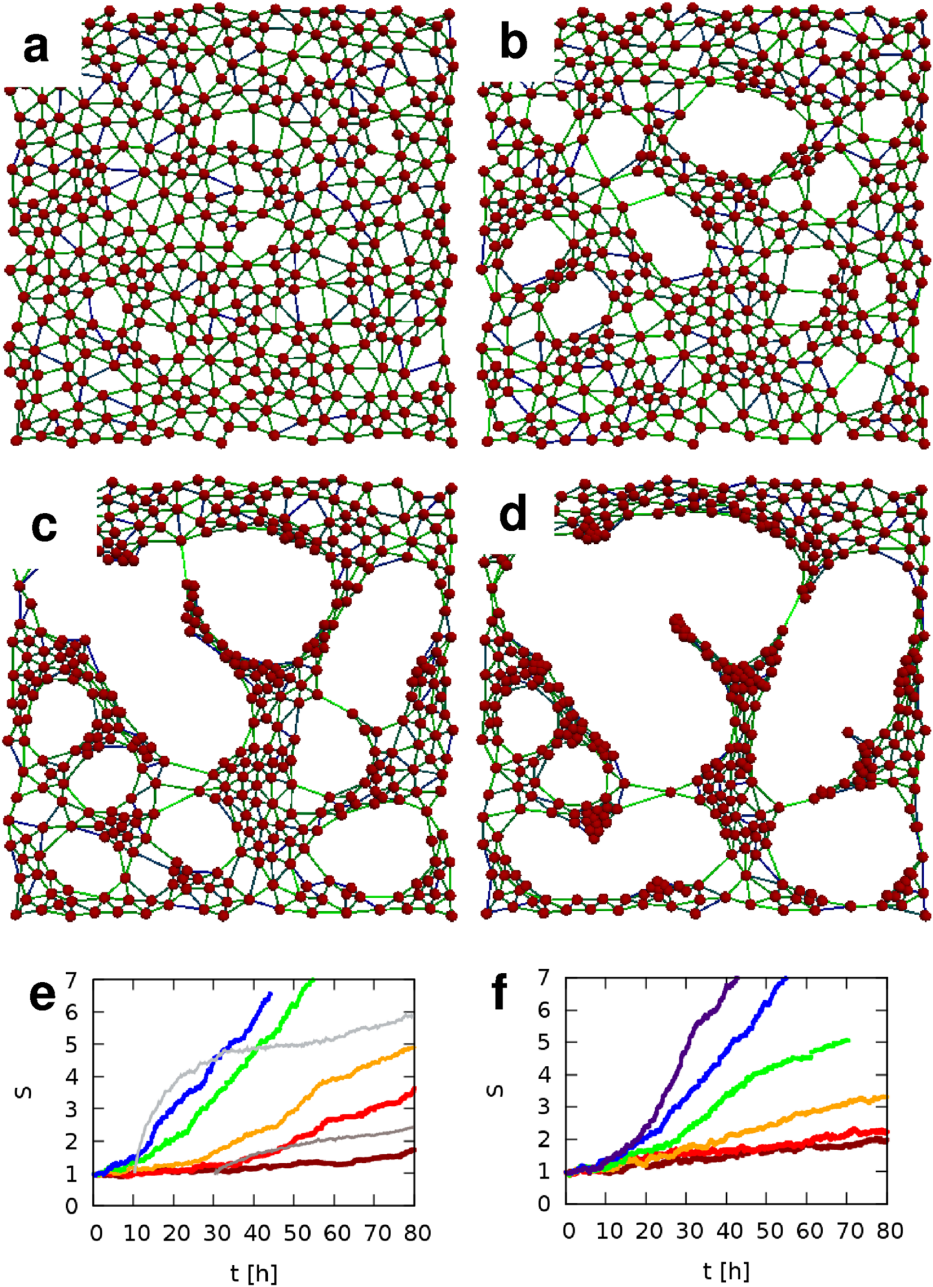
Time development of a typical simulation, at $\hbox {t}=2.5$ h (**a**), $\hbox {t}=7.5$ h (**b**), $\hbox {t}=13$ h (**c**) and $\hbox {t}=16$ h (**d**). Key simulation parameters are $F^*/F^0=2$, and $\alpha =0.1$—others are specified in the Supplement. (**e**, **f**) The progress of patterning is characterized by the standard deviation of particle density, *S*, as a function of time. Pattern formation is faster for higher target force $F^*$ values (**e**) and for higher values of $\alpha$, the motion bias towards external forces (**f**). Red to blue colors indicate values of 0.5, 0.75, 1, 2, and 4 for parameter $F^*/F^0$ (**e**), and 0.001, 0.003, 0.01, 0.03, 0.1, 0.3 for parameter $\alpha$ (**f**), respectively. Each curve is an average calculated from $n=4$ independent stochastic simulations. As a comparison, *S*(*t*) curves are also shown for SPC111 (dark gray) and VMC20 (light gray) cell cultures (**e**).

**Figure 7:**
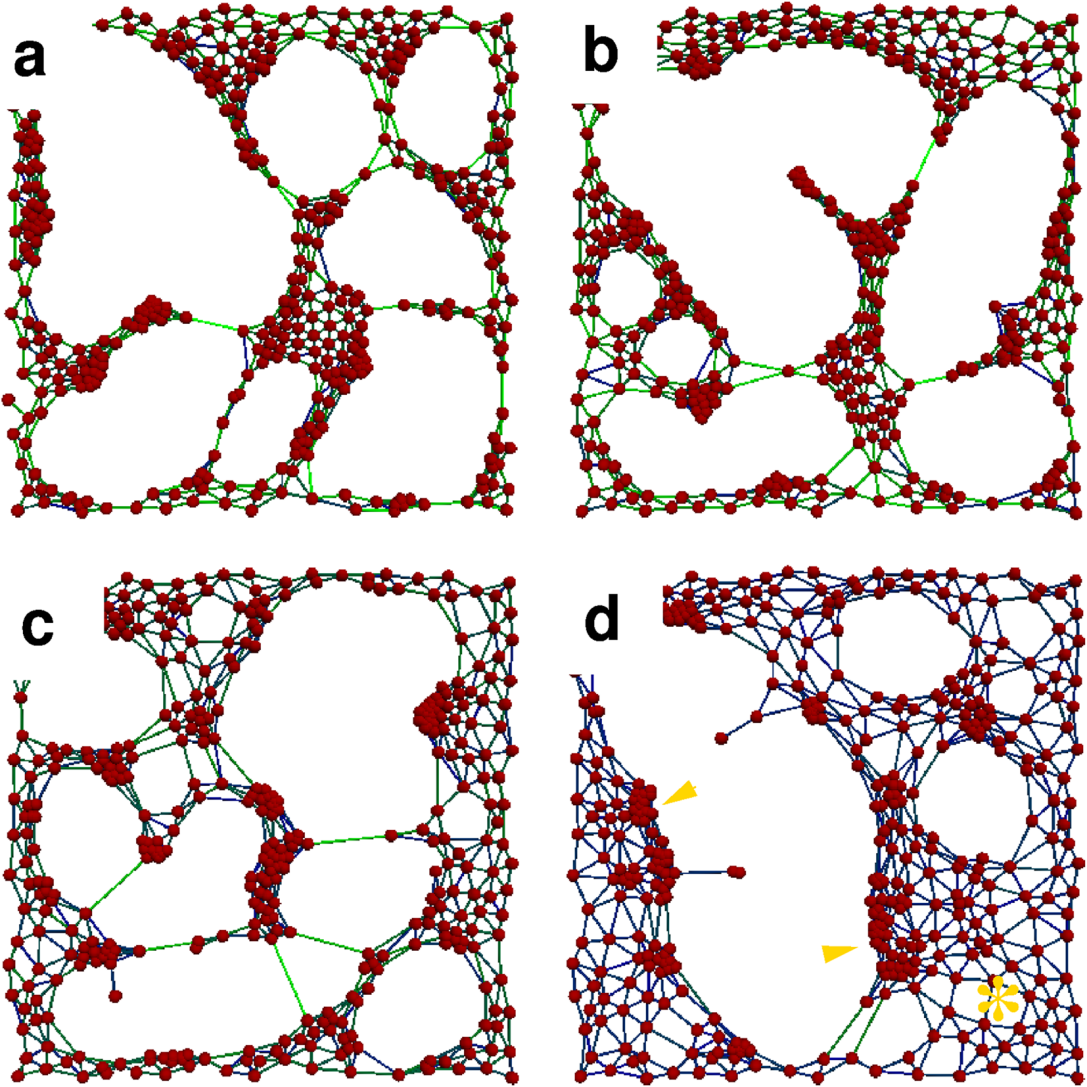
Morphologies characteristic for various target force values. Simulations performed with $F^*/F^0=4$ (**a**), $F^*/F_0=2$ (**b**), $F^*/F^0=1$ (**c**), and $F^*/F^0=0.5$ (**d**) are shown at the same stage of pattern formation ($S=3.85$). For small forces $F^* < F^0$ high cell density clusters develop at the boundary of cell free areas (arrowheads), while the particle density remains low far from such boundaries (asterisk). In contrast, for large forces $F^* > F^0$ the particle density is more uniformly high (**a**).

**Figure 8:**
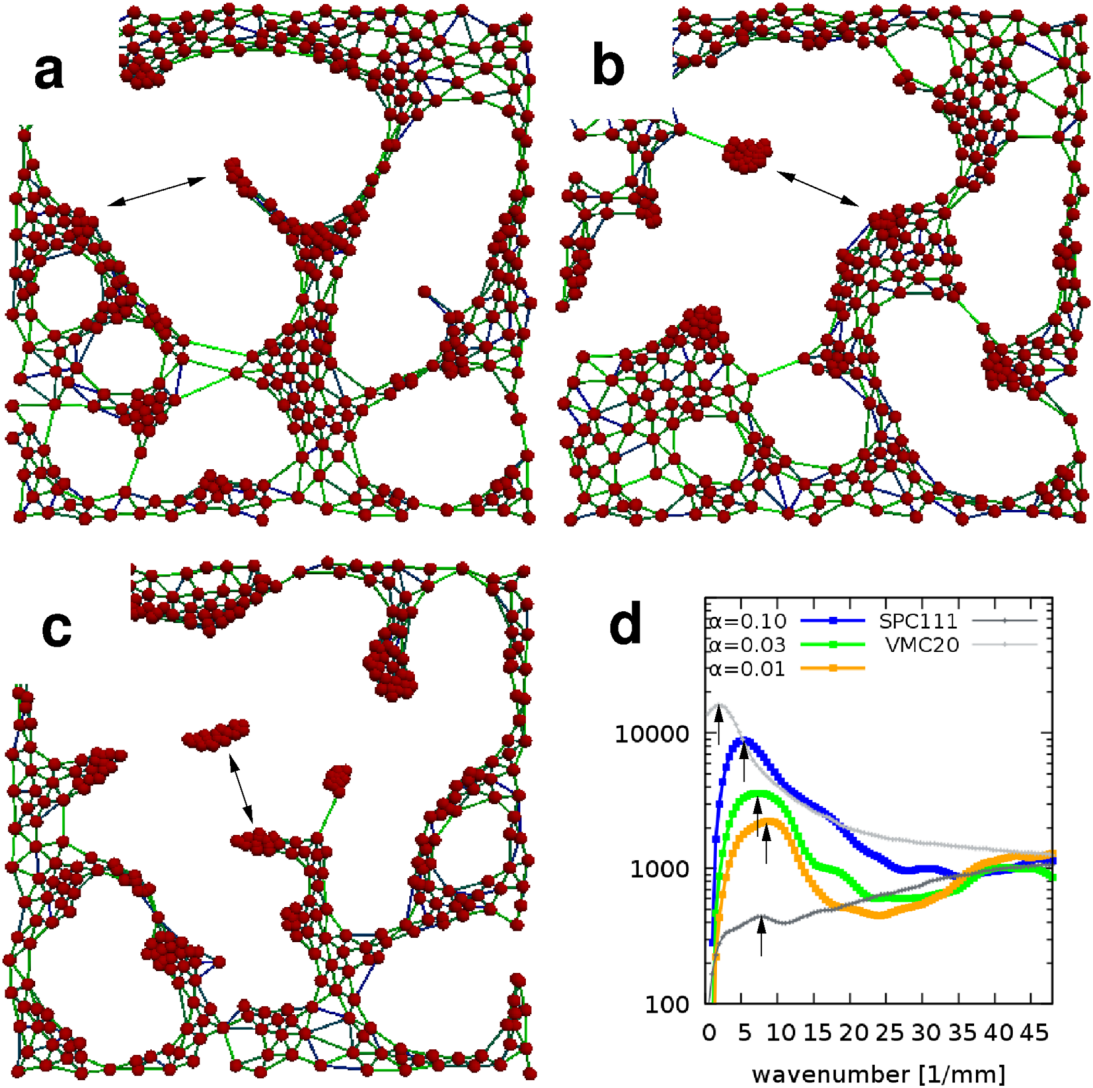
Morphologies characteristic for various adhesion parameter values. Simulations performed with $\alpha =0.1$ (**a**), $\alpha =0.03$ (**b**), and $\alpha =0.01$ (**c**) are shown at the same stage of pattern formation ($S=3.5$). The characteristic pattern size is set by the stability of cell-substrate adhesion as the spatial power spectra of the configurations indicate (**d**). For smaller values of $\alpha$, the size of the aggregates and the characteristic distance between aggregates (arrows) decreases. Light gray and dark gray lines represent radial power spectra calculated from phase contrast images of VMC20 and SPC111 cell cultures on the seventh day in vitro.

To better understand how cell contractility can create the MPM nodules observed in cell culture experiments, we constructed a computational model of cell layer mechanics, utilizing recent modeling methods^28,29^. The model is driven by stochastic changes in cell-cell contacts, and is based on a Gillespie simulation^30^. The purpose of the model is to explore how the diversity of MPM nodule types (Figs. 1, 2) are determined by cellular features like substrate adhesion, contractility or cell crowding.

Simulations of a two dimensional contractile monolayer (see Supplementary Material and Fig. 6) reveal a pattern formation process analogous to the behavior of cultured MPM cells (Fig. 4). First, as we demonstrate in the Supplementary Material Section 3, the initial inhomogeneity of the monolayer is amplified resulting in the formation of discontinuities or cell-free areas. Next, the cell-free areas continue to expand because their boundaries are unstable: links constituting the boundaries balance the pulling forces exerted by cells within the bulk of the monolayer. Thus, connections at boundaries experience higher tensile stress which increases both their length and the probability of their removal. Both effects expand the empty area. As a consequence, cell density increases in cell-populated areas, which progressively begin to resemble actual MPM contractile nodes connected by multicellular linear strands. While nodules develop in most MPM cultures (Figs. 1, 2), the emergence of cell-free zones is not observed in all MPM lines. When nodules form on top of a basal monolayer, our model represents the cell population that was excluded from the monolayer. Thus, we assume that when a monolayer is present, it functions as a substrate for the aggregating cells.

During the aggregation process, the spatial distribution of cells becomes less homogeneous, and the progress of patterning was characterized by the standard deviation of the coarse-grained particle density field (*S*). While the time course shown in Fig. 6 is characteristic for all simulations, two model parameters, $F^*/F^0$ and $\alpha$, have important roles in determining both the morphology and the speed of the aggregation.

The ratio between the steady state contractile force of the cells, $F^*$, and the Bell threshold^31^ of adhesion stability, $F^0$ determines the speed of aggregation. Experimental *S*(*t*) data are comparable with those obtained from simulations: SPC111 and VMC20 cultures are best described with a $F^*/F^0$ ratio of 0.75 and 4, respectively. Decreasing contractility results in slower patterning (Fig. 6e), in accord with experimental data obtained with the ROCK inhibitor (Fig. 4c,f). Figure 7 compares morphologies that are at the same, late stage ($S \approx 4$) of the patterning process for various values of $F^*/F^0$. For small forces $(F^* < F^0)$, cells accumulate at the boundary of cell-free areas, similar to the in vitro patterns observed for the Meso80 and Meso53 lines (Fig. 2). In this case, the aggregation process is slower—again, in accordance with empirical in vitro data. For large forces ($F^* > F^0$), links with high particle density interconnect similarly dense nodules, resembling the patterns observed in SPC111 cultures (Fig. 1).

Model parameter $\alpha$, characterizing the magnitude of external force-directed cell displacements, sets the spatial scale of the pattern as predicted by Supplementary Eq. (18) (Fig. 8). When cell-substrate adhesion sites are stable ($\alpha \ll 1$), smaller clusters develop which are close to each other. In contrast, when cells respond strongly to external forces (i.e., substrate adhesion is weak or highly adaptable), fewer and larger clusters form. Thus the proximal aggregates in SPC111 cultures are consistent with more stable cell-substrate adhesion ($\alpha \approx 0.01$), while the more distant aggregates of VMC20 cells indicate less stable adhesion complexes ($\alpha \approx 0.3$).

## Discussion

Aggregation and sorting of zebrafish cells was shown to involve acto-myosin contractility within the cortical cytoskeleton^32^. Here, we show that cell groups may also contract through stress cables spanning across several cells, and the resulting system self-organizes into expanding cell-free areas and eventually into free-standing aggregates. Similar behavior also takes place in a highly melleable environment, such as a Matrigel gel^29^ and also at much smaller length scales in the cytoskeleton of individual cells, where acto-myosin contractility gives rise to f-actin bundles. For example, a similar contractile system has been studied in the context of pattern formation within the actomyosin cell cortex^33^. Further extensions of this concept have been described recently^34,35^.

Just as with cell-cell connections, mechanical load acting on cell-substrate adhesion complexes reduces their lifetime^36^. To relate cell movements and external forces acting on a cell we envision the following process: when a cell-substrate connection breaks, the same mechanical load is distributed along the remaining adhesion complexes. Thus, each of the remaining adhesion sites transmits a larger force, their strain is increased leading to a small displacement of the cell body in the direction of the net external force acting on the cell. Furthermore, when new adhesion complexes form, their equilibrium (stress-free) configuration will correspond to the actual, slightly shifted position of the cell. Thus, by detaching and re-attaching adhesion complexes, the cell relaxes the shear stress between its cytoskeleton and the adhesion substrate, and moves in the direction of the external force. In addition to this purely mechanical connection, external stress may also effect the polarity of active cell motility^37,38^.

Our results may let us hypothesize the following sequence of events for mesothelioma progression. The slow proliferation of MPM tumor cells initially remain hidden as their density and contractility remains below the instability threshold Supplementary Eq. (19). When the cell density or their abnormal contractility exceeds the patterning threshold, suddenly nodules appear at multiple locations. The nodules become progressively more contractile as the local cell density increases, and they may even internalize ECM, blood vessels or cells of the underlying stroma. We hypothesize that contractility may also promote pinching off the nodules into the pleural space and thus contribute to local spreading of the disease.

Cancer stem cells facilitate tumor growth and metastasis^39^. Cancer stem cells enhance the ability of tumors to grow under anchorage independent conditions and as spheroids in suspension cultures. ROCK inhibitors have been reported to inhibit the contractility and invasive potential of cancer stem cells^40^. We demonstrate for the first time that ROCK inhibitors affect the formation of MPM nodules by influencing the contractility of the actin/myosin filaments. Future work will be necessary determine the role of cancer stem cells in affecting nodule formation and contractility.

Previous investigations particularly in non-tumorous cells described an IC50 lower than $2\mu$M for both blebbistatin and Y27632^26,41^. However, several recent preclinical studies in different tumor entities used significantly higher concentrations of those inhibitors to achieve meaningful effects^42–45^. Furthermore, the concept of multicellular resistance suggests that tumor cells are more resistant to drugs when they are part of multicellular aggerates, presumably due to limited intake within the bulk of the 3D structure^46–48^. Accordingly, without affecting cell viability but to achieve similar effects as in an 2D environment, we considered and applied higher concentrations of blebbistatin and Y27632 to our 3D preclinical model in MPM.

Our data demonstrate that MPM cells have the capacity to form multicellular nodules by cell contractility-derived forces in vitro. We found that nodule formation is significantly less common in non-MPM tumor cell lines. Nevertheless, a few of those epithelial-origin tumor cell lines also formed nodules in vitro. Accordingly, in vitro nodule formation is more characteristic but neither specific nor exclusive for MPM. Among those non-MPM tumor cell lines that form nodules, similar biological and molecular processes might be underlying their tumor nodule formation. Further investigations will be necessary to identify similarities and differences across different nodule forming tumor cell lines. While traditional pharmacological intervention focus on the altered biochemistry of a single cancer cell, the multicellular aspects of a growing tumor are of substantial medical relevance^49^. Yet, tumor morphogenesis is a complex, tissue-scale process similar to embryonic development. Better understanding of this complexity may open a pathway for treatment modalities substantially different from the current anti-proliferative agents. The presented experimental model could provide a tool to test various treatment modalities in which tissue deformation and morphology recapitulate nodule formation in MPM specimens. However, further investigations will be necessary elucidate the growth dynamics of in vitro nodule formation.

Our study indicated the presence of multicellular actin cables suggesting that MPM cells might use a mechanosensing-based long-range communication mechanism within the mechanically interlinked tumor nodules; a process reminiscent of multicellular stress cable-connected cells during Drosophila embryogenesis^50^. The cellular contractile activity, we document here, may exert forces that can lead to the internalization of parts of the host tissue environment including preexisting blood vessels^51^. Efficient inhibition of nodule formation could enhance the effective drug concentration at the targeted cells, because cells within poorly vascularized 3D nodules experience lower levels of systemically-administered drugs. Myosin inhibitors were previously used in pre-clinical studies, albeit with a different concept of their mechanism of action^52,53^. Still, interfering with actomyosin contractility has emerged as a novel therapeutic approach in various malignancies. In this regard, migrastatics, a novel term for drugs mainly interfering with actomyosin contractility like myosin inhibitors may provide many opportunities in targeting cancer cell invasion and metastasis^5^. In our study, the hereby demonstrated ability of myosin-II inhibitors to disperse MPM nodules may underline a rational for targeting actomyosin contractility in MPM. Nevertheless, future translational studies will be essential to better understand in vitro nodule formation as target in MPM.

## Methods

### Cell lines

SPC111 cell line was obtained from Sigma. SPC212 and M38K cell lines were established from human biphasic MPMs, kindly provided by Prof. R. Stahel (SPC212, University of Zurich, Zurich, Switzerland) and Prof. V.L. Kinnula (M38K, University of Helsinki, Helsinki, Finland), respectively. The MPM P31cis cells and I2 cells were a kind gift from Prof. K. Grankvist (University of Umea, Sweden) and Prof. A. Catania (University of Milano, Italy), respectively. The VMC6, VMC12, VMC20, VMC23, VMC40, VMC48, Meso49, Meso53, Meso62, Meso71, Meso80, Meso84, Meso92, Meso103, Meso110, Meso161, Meso189, Meso194, Meso200, Meso204, Meso205, Meso208, Meso221 cell lines, the VM15 and VM47 melanoma cell lines were established at the Medical University of Vienna as described earlier^54,55^. The small cell lung cancer cell line HLHE was provided by Sabine Spiegl–Kreinecker (Neurocampus, Kepler University, Linz). NP3 normal mesothelial cells were isolated from pneumothorax patients^56^. The non-MPM tumor cell lines MEWO, A375, DLD-1, HT29, SW1417, HCT116, A549, CRL5922, CRL2066, H1650, U87, U373, A172 and T98 were purchased from the ATCC. The GBM1 glioblastoma cell line was established as previously described^57^. The HCA7 colorectal carcinoma cell line was purchased from the ECACC. The M24met melanoma cell line was kindly provided by BM. Mueller (Scripps Research institute, La Jolla, CA, USA). Cell lines were authenticated by short-tandem-repeat DNA profiling. All cell lines were tested for mycoplasma contamination using the MycoSensor PCR Assay Kit 302108 (Agilent). Genetical characterization of MPM cell lines was carried out by identification of alterations in TERT promoter region, BAP1, TP53 and NF2 described in^22^.

### Culture conditions

Cells were grown at $37^o\hbox {C}$ in a humidified, 5% $\hbox {CO}_2$, 95% air atmosphere. Dulbecco’s Modified Eagle Medium (DMEM, Lonza) containing L-glutamine was supplemented with 10% fetal bovine serum (Invitrogen) and penicillin-streptomycin-amphotericin B (Lonza). MPM cells were cultured in standard culture dishes. NP3 cells were cultured in gelatin-precoated dishes, for up to three passages. Gelatin-coated dishes were obtained by incubating 10% gelatin solution-B (Sigma) in PBS for 45 min at room temperature. We also prepared 1.7 mg/ml Collagen-I (Corning) and Matrigel (ECM gel, Sigma) gel substrates according to the manufacturer’s instructions. To visualize remodelling of these substrates, we mixed fluorescent latex beads ($0.5 \,\mu \hbox {m}$, sulfate-modified polystyrene, Sigma) into the gel-forming solution.

### Surgical specimen

Frozen sections from MPM tumors were obtained from the Biobank of the Department of Thoracic Surgery, Medical University of Vienna. All patients provided informed consent for the collection and use of tumor material for scientific purposes. The study was approved by the Ethic Commission of the Medical University of Vienna (#904/2009).

### Orthotopic in vivo MPM xenograft model

8-week-old male SCID mice were obtained from the animal facility of the Ist Department of Pathology and Experimental Cancer Research of Semmelweis University. The animal study protocols were conducted according to National Institute of Health (NIH) guidelines for animal care and were approved by the Animal Care and Use Committee of Semmelweis University and NÉBIH (PEI/001/2457-6/2015). 2 million SPC111 cells in $100 \,\mu \hbox {l}$ DMEM were inoculated into the chest cavity using a protocol modified after^58^. Under anesthesia (Ketamine-Xylazine, 80:12 mg/kg, Cat. No.: K113, Sigma Aldrich) a midline incision was made on the chest and muscles on the right side were separated and 2 million cells were injected between the 2nd and 3rd cartilages. Following 21–35 days animals were sacrificed and the chest cavity was photographed. Finally, MPM tumor nodules were harvested and stored as snap-frozen tissue or formalin-fixed and paraffin-embedded.

### Reagents

To interfere with normal actomyosin function, we utilized Y27632, the rho kinase (ROCK) inhibitor (Merck Millipore) and Blebbistatin (Merck Millipore), an inhibitor of actomyosin crosslinking. Y27632 was dissolved in distilled water, as a 10 mM stock solution, and used at $50\hbox {-}100 \,\mu \hbox {M}$ final concentrations in DMEM. Blebbistatin was dissolved in DMSO, stored as 50 mM aliquots, and used at $40\hbox {-}80 \,\mu \hbox {M}$ final concentrations. Control cultures were treated with identical amount of DMSO as drug treated cultures. Hydroxyurea (Sigma-Aldrich), an inhibitor of deoxyribonucleotide production was applied to inhibit cell proliferation. Hydroxyurea was dissolved in distilled water, as a 50mg/ml stock solution, and used in the $15\hbox {-}1000 \,\mu \hbox {M}$ concentration range.

### Immunostaining

SPC111 cells were seeded at confluent density ($40000\hbox { cells/cm}^2$) either on the surface of collagen gels or on glass coverslips and grown for 6-11 days. Samples were fixed using 4% paraformaldehyde for 15 min at $4^o\hbox {C}$, either permeabilized with 0.25% Triton-X100 for 10 min at $4^o\hbox {C}$, and incubated with polyclonal anti-beta-catenin antibodies (1:100, C2206, Sigma) or blocked in complete blocking buffer (0.5% BSA, 0.1% TRiton X-100, 5% FBS in PBS), and incubated with anti-myosin light chain phospho (S20) antibody (1:200, ab2480, abcam). After washing, sections were incubated for 30 min either with anti-rabbit Alexa-488 secondary antibody (Life Technologies) for anti-beta-catenin primary antibody, or anti-rabbit Alexa555 secondary antibody (Thermo Scientific) for anti-phospho MLC antibody. Filamentous actin was stained either by Phalloidin-TRITC (P1951, Sigma), or Phalloidin-FITC (P5282, Sigma). Nuclear staining was performed by ProLong Glass Antifade Mountant with NucBlue Stain (P36983, Thermo-Fisher).

For immunofluorescence analysis of whole-mount samples, animals were sacrificed by anesthetic overdose by Ketamine-Xylazine (Sigma Aldrich). The diaphragm was fixed by injecting 2% paraformaldehyde at $4^o\hbox {C}$ into the abdomen and into the thorax, 2 ml and 1 ml, respectively. After 15 min, diaphragm was removed and washed in PBS. The sample was permeabilized with 1,25% Triton-X 100 for 15 min, at room temperature. After washing, samples were incubated overnight with primary antibody (polyclonal anti-beta-catenin 1:100, C2206, Sigma). After 8 hours of washing in PBS, samples were incubated with donkey anti-rabbit alexa fluor 488 and TRITC-phalloidin for 4 hours. After another 8 hours of washing samples were put onto slides, covered with fluorescence mounting medium (DAKO). The human samples were treated in the same way. Samples were analyzed by confocal laser scanning microscopy using either the Bio-Rad MRC-1024 system (Bio-Rad) or the Zeiss LSM-710 system (Zeiss).

### Physical sections

Vibratome sections, $100 \,\mu \hbox {m}$ thick, were obtained from SPC111 cell cultures grown on the surface of collagen-I gels. Samples were embedded in 3% acrylamide–1.5% agarose mixture. Sections were cut perpendicularly to the surface of the collagen-I gel and stained post-sectioning with 0.5% Toluidin blue.

For semithin sections, tumor bearing animals were anesthetized as mentioned above and perfused via the left ventricle with PBS for 10 min and with a mixture of 4% paraformaldehyde and 1% glutaraldehyde in PBS (pH 7.2) for 15 min at room temperature. Diaphragms with tumors were removed, cut into 1–2 mm pieces and immersed in the same fixative for an additional 2 h. Pieces were postfixed in 1% OsO4 and 0.5% K-ferrocyanide in PBS for 2 h, dehydrated in a graded series of acetone and embedded in Spurr’s mixture. Semithin sections were cut by an RMC MT-7 ultramicrotome and stained by 0.5% toluidine blue at pH 8.5. Tissue culture samples were treated by the same fixatives. After dehydration the samples were treated by propylene oxide, which resulted in the separation of the cultured cells in sheets from the bottom of the flasks. Embedding and sectioning was performed as indicated above.

### Time-lapse microscopy

As described in^59^, time-lapse recordings were performed on a computer-controlled Leica DM IRB inverted microscope equipped with a Marzhauser SCAN-IM powered stage and a 10x N-PLAN objective with 0.25 numerical aperture and 5.8 mm working distance. The microscope was coupled to an Olympus DP70 color CCD camera. Cell cultures were kept at $37^o\hbox {C}$ in humidified 5% $\hbox {CO}_2$ atmosphere in a stage-top mini incubator during imaging. Phase contrast images of cells were collected consecutively every 10 minutes from each of the microscopic fields.

To maintain high cell density cultures for several days, we restricted cell spreading. Our cell culture utilized three, 6 mm diameter mini-wells, fusion-deposition (“3D”) printed from polylactic acid (PLA) into standard 35 mm tissue culture dishes (Greiner)^60^. As cells covered only 10% of the culture area, this configuration permitted to achieve high cell densities ($10^5/\hbox {cm}^2$) with a high medium/cell ratio. The 3 ml medium was replaced every 5 days. Four modified culture dishes, each containing 3 wells, were observed by time-lapse microscopy for up to 14 days. We recorded four adjacent fields from each well, thus we collected 48 images in each imaging cycle.

### Monitoring of the aggregation process

First, we determined the overall cell movement directionality using an optical flow (PIV) method^24^. We obtain a velocity field for each consecutive pair of images by determining the displacements of image details. We then placed tracer particles on a uniform grid onto the first frame of an image sequence. The location of the tracer particles were updated using the estimated optical flow-derived velocity at the corresponding location, estimated by linear interpolation^24^. Thus, movement of the tracer particles mimicked that of the multicellular structures in the experiment.

Contractile activity was also charactized by the divergence of the velocity field, a technique used previously to study cardiomyocyte phenotype in vitro^23,27^. The optical flow-based method does not distinguish between cell contraction and cell free area expansion. Thus, to identify contractile centers, we estimated the negative divergence of the optical flow field, which we termed convergence^23^.

## Supplementary information

Supplementary Movie S1.

Supplementary Movie S2.

Supplementary Movie S3.

Supplementary Movie S4.

Supplementary Movie S5.

Supplementary Information.
