# Supplementary material for: Multicellular contractility contributes to the emergence of mesothelioma nodules
Source: Sci Rep. 2020 Nov 18;10:20114. doi: 10.1038/s41598-020-76641-x (PMC7675981; doi:10.1038/s41598-020-76641-x)
Supplement: Supplementary file 6 — Supplementary Information. [file 41598_2020_76641_MOESM6_ESM.pdf]

Multicellular contractility contributes to the emergence of mesothelioma nodules.

Julia Tarnoki-Zach[1], Paul Stockhammer[2,3], Dona Greta Isai[4], Elod Mehes[1], Balint Szeder[5], Ildiko Kovacs[6], Edina Bugyik[7], Sandor Paku[7], Walter Berger[8], Sufi Mary Thomas[9], Zoltan Neufeld[10], Balazs Dome[3,6,11,12], Balazs Hegedus[2], and Andras Czirok[1,4,*]

[1] Department of Biological Physics, Eötvös University, Budapest, Hungary
[2] Department of Thoracic Surgery, Ruhrlandklinik, University Duisburg-Essen, Essen, Germany
[3] Division of Thoracic Surgery, Department of Surgery, Comprehensive Cancer Center, Medical University of Vienna, Austria
[4] Department of Anatomy & Cell Biology, University of Kansas Medical Center, Kansas City, KS, USA
[5] Institute of Enzymology, Research Centre for Natural Sciences, Budapest, Hungary
[6] National Korányi Institute of Pulmonology, Budapest, Hungary
[7] First Department of Pathology and Experimental Cancer Research, Semmelweis University, Budapest, Hungary
[8] Institute of Cancer Research and Comprehensive Cancer Center, Department of Medicine, Medical University of Vienna, Austria
[9] Department of Otolaryngology, University of Kansas Medical Center, Kansas City, KS, USA
[10] School of Mathematics and Physics, University of Queensland, Brisbane, Australia
[11] Department of Thoracic Surgery, Semmelweis University and National Institute of Oncology, Budapest, Hungary
[12] Department of Biomedical Imaging and Image-guided Therapy, Division of Molecular and Gender Imaging, Medical University of Vienna, Vienna, Austria
*aczirok@gmail.com

Supplemental Material

Computational model

To explore the dynamics of cell contraction-driven aggregation and nodule formation, we adapted our cell-resolved elasto-plastic computational framework [2, 4] into a two dimensional model. The source code of our simulations is available at http://github.com/aczirok/cellmech. Briefly, forces distributed along the membranes of mechanically coupled adherent cells are replaced by a single net force and a torque, simplifying the system to a network of particles and elastic beams.

In the computational model, torques and shear forces arise due to relative movement of adjacent particles. Hooke's law determines the force $F^{\parallel}$ needed to uniaxially compress or stretch cells. In particular, $F_l^{\parallel}$ is associated with changing the length of link l which connects particles i and j located at $\mathbf{r}_i$ and $\mathbf{r}_j$, respectively:

$$F_l^{\parallel} = k(|\mathbf{r}_j - \mathbf{r}_i| - \ell_l). \tag{1}$$

In Eq. (1) ℓ_l is the equilibrium length of link l and $k > 0$ is a model parameter, characterizing the stiffness of the cytoskelon.

A link l exerts a torque if its preferred direction at particle i, $\mathbf{t}_{i,l}$, is distinct from its actual end-to-end direction $\mathbf{u}_{ij}$. We assume that the torque exerted on particle i is proportional to the difference between the preferred and actual directions:

$$\mathbf{M}_{i,l} = g(\mathbf{t}_{i,l} \times \mathbf{u}_{ij}), \tag{2}$$

where the microscopic bending rigidity g, a model parameter, can be calibrated from macroscopic material properties of the tissue [2]. The condition for mechanical equilibrium, together with Eqs (1) and (2) allow the calculation of the forces and torques within the system [2].

Multicellular plasticity is modeled using rules that rearrange the network of intercellular adhesions. The stability of intercellular adhesion complexes depends on the tensile forces they transmit [9]. Thus, in our model the probability of removing link l during a short time interval Δt follows Bell's rule [1] as

$$p_l \Delta t = A e^{F_l^t / F^0} \Delta t, \tag{3}$$

where A is a scaling factor which characterizes the stability of connections, F^0 is a threshold value and F_l^t is the tensile component of the force transmitted by the link.

Mechanical connections can be established between two Voronoi neighbor particles, i and j. We assume, that during a short time interval, Δt, the probability of inserting a new link is a decreasing function of the distance $d_{i,j}$ between the particles:

$$q_{i,j}\Delta t = B\left(1 - \frac{d_{i,j}}{d_{max}}\right)\Delta t. \tag{4}$$

The scaling factor B represents the intensity of cellular protrusive activity devoted to scanning the environment and the ability to form intercellular contacts. The maximal distance cells explore for new connections is denoted by d_{max}.

Simulations are event-driven: using the probability distributions (3) and (4), we generate the next event μ and waiting time τ according to the stochastic Gillespie algorithm [3]. The waiting time until the next event is chosen from the distribution

$$\log P(\tau) = -\tau\left(\sum_l p_l + \sum_{i,j} q_{i,j}\right), \tag{5}$$

where the sums are evaluated by iterating over existing links l as well as over all possible Voronoi neighbor particle pairs i, j not connected by a link.

Mechanical model of a contractile cell layer

We assume that cells maintain a specific contractile environment by increasing or decreasing their contractility if the tensile stress within the monolayer is below or above a target value, σ^*, respectively. Thus, the mechanical stress within the monolayer is a combination of the passive elastic stresses and the active stress generated by acto-myosin contractility. For simplicity, we consider such a system in one dimension. The time development of $\sigma(x,t)$, the local mechanical stress at position x at time t is given by

$$\frac{\partial \sigma}{\partial t} = \tilde{E}\rho\frac{\partial v}{\partial x} - \tilde{C}(\sigma - \sigma_*\rho) \tag{6}$$

where $\tilde{E}\rho$ is the elastic modulus of a pile of cells crowded at a density ρ and $\tilde{C}$ is the rate of homeostatic stress adjustment. The first term on the right hand side of (6) represents elastic stress and the second term describes the homeostatic maintenance of a contractile state where each cell experiences a tensile stress σ_*.

The mechanical equilibrium of surface-attached cells requires the net force exerted by adjacent cells to be balanced by the force transmitted via cell-substrate adhesion. Cells move through a series of complex biochemical processes, which – through largely unexplored mechanisms – can be guided by external forces acting on a cell. Xenopus mesendoderm cells, for example, were shown to move against an external pulling force [7]. Neutrophils have been shown to be guided by the shear stress within the boundary layer of the flowing medium [5]. Cell rearrangements within a confluent monolayer were suggested to follow local orientations of the maximal principal stress [6]. Here we propose

that MPM cells tend to move in ' the direction of the net external force acting on them as

$$v = \tilde{\alpha}\frac{\partial \sigma}{\partial x}. \tag{7}$$

While Eq. (7) is formally analogous to a viscous drag, in this system it represents mechanical stress guided bias within the active movement of cells.

Finally, in the absence of cell death and proliferation the cell density $\rho(x,t)$ satisfies the conservation equation

$$\frac{\partial \rho}{\partial t} + \frac{\partial}{\partial x}(v\rho) = \tilde{D}\frac{\partial^2 \rho}{\partial x^2} \tag{8}$$

where the diffusion term on the right-hand-side accounts for random movements of the cells.

Patterning instability

To explore the emergent patterning mechanism specified by Eqs. (6) – (8), we performed a linear stability analysis of a stationary state $v(x,t) = 0$ with uniform cell density $\rho(x,t) = \rho_0$ being at the homeostatic tensile stress $\sigma(x,t) = \sigma_* \rho_0$. To probe the stability of such a uniform monolayer, we consider the time evolution of small perturbations of the form

$$\rho(x,t) = \rho_0 + \hat{\rho} e^{st+iqx} \tag{9}$$

$$v(x,t) = \hat{v} e^{st+iqx} \tag{10}$$

$$\sigma(x,t) = \sigma_0 + \hat{\sigma} e^{st+iqx} \tag{11}$$

where s is the growth rate of a monochromatic perturbation of wavenumber q, assuming that the perturbation amplitudes $\hat{\rho}, \hat{v}, \hat{\sigma}$ are small.

After substituting the perturbed fields into Eqs. (6) –(8) and neglecting higher than linear terms we obtain

$$s\hat{\rho} + iq\rho_0\hat{v} = -q^2\tilde{D}\hat{\rho} \tag{12}$$

$$s\hat{\sigma} = i\tilde{E}\rho_0 q\hat{v} - \tilde{C}(\hat{\sigma} - \sigma^*\hat{\rho}) \tag{13}$$

$$iq\hat{\sigma} = \hat{v}/\tilde{\alpha} \tag{14}$$

Eliminating the amplitudes yields the following quadratic equation for the growth rate s

$$s^2 + s\left(\tilde{C} + q^2\tilde{E}\rho_0\tilde{\alpha} + \tilde{D}\right) + q^2\left[q^2\tilde{E}\rho_0\tilde{\alpha} + C - \sigma^*\rho_0\tilde{C}\tilde{\alpha}\right] = 0 \tag{15}$$

with the solutions

$$s_\pm(q) = \frac{1}{2}\left(-b(q) \pm \sqrt{b(q)^2 - 4q^2 a(q)}\right) \tag{16}$$

where

$$a(q) = \left[q^2\tilde{E}\rho_0\tilde{\alpha} + C\right]\tilde{D} - \sigma^*\tilde{C}\rho_0\tilde{\alpha}, \quad b(q) = \tilde{C} + q^2\left(\tilde{E}\rho_0\tilde{\alpha} + \tilde{D}\right). \tag{17}$$

When $a(0) > 0$ then $a(q)$ is positive for all wavenumbers, and the eigenvalues are either real and negative, or complex conjugate pairs with negative real part. In this case the uniform steady state is stable and small density perturbations decay. For $a(0) < 0$ there is a band of wavenumbers $0 < q < q_{max}$ where one of the eigenvalues is positive, rendering the uniform state is unstable and thus allowing the formation of cell aggregates. The range of instability is bounded by

$$q_{max}^2 = \frac{\tilde{C}}{\tilde{E}\rho_0\tilde{\alpha}} \left(\frac{\sigma^* \rho_0 \tilde{\alpha}}{\tilde{D}} - 1 \right), \tag{18}$$

which sets the characteristic scale of the structures as $\sim q_{max}^{-1}$. The condition for the instability in terms of the model parameters can be written as

$$\sigma^* > \frac{\tilde{D}}{\tilde{\alpha}\rho_0} \tag{19}$$

showing that the stability threshold increases with the diffusivity $\tilde{D}$ and decreases with the density and the ability of the cells to respond to mechanical cues $\tilde{\alpha}$. Thus, when the density of cells with a steady contractility reaches a threshold value, our analysis predicts that the monolayer will break up and cells will form aggregates. The typical scale of the developing pattern is set by $1/q_{max}$, thus cells that are less responsive to external forces, or with more stable adhesion to the substrate (i.e. characterized by lower values of $\tilde{\alpha}$) are expected to form smaller aggregates. Similarly, smaller aggregates are expected for cells that are more contractile.

Cell-resolved simulations of a contractile monolayer

While the above analysis helps us chart the generic features of the patterning instability, to fully explore the consequences of the self-tensioned monolayer model, we perform simulations with a cell-resolved particle model. We augmented our particle model of elasto-plastic cell assemblies (see Methods and [2]) with features corresponding to Eqs. (6) and (7).

We assume that model particles maintain a certain tensile force F^* in each link by adjusting the equilibrium link length ℓ_l in Eq. (1). In analogy with Eq.(6), the rate of change in the link length is proportional to the difference between F^* and F_l^t, the tensile component of the force transmitted by the link:

$$\frac{d\ell_l}{dt} = Cd_0 \frac{F_l^t - F^*}{F^*} + D\mu_l(t), \tag{20}$$

where $1/C \approx 1$ h sets the temporal scale of the feedback regulation. The last term in (20) is a normalized white noise, representing random cell shape changes due to factors not considered explicitly in our model. Parameter $D \approx 1\mu\text{m}^2/h$ controls the noise amplitude. Equation (20) was integrated over a time interval τ, elapsed until the next stochastic change in the connectivity (adjacency) of the particles.

As we explicitly represent mechanical connections between adjacent cells, we will also introduce model objects that represent adhesion between the cells (particles) and the substrate. Thus, for each particle i we introduce its equilibrium position $\mathbf{r}_i^0$ and equilibrium orientation ϕ_i^0. The force and torque transmitted to the substrate are given by the linear relations

$$\mathbf{F}_{i,0} = k_0(\mathbf{r}_i - \mathbf{r}_i^0), \quad \mathbf{M}_{i,0} = g_0(\phi_i - \phi_i^0)\mathbf{e}_z. \tag{21}$$

The stress-guided motility drift (7) is introduced as

$$\frac{d\mathbf{r}_i^0}{dt} = \alpha \frac{Cd_0}{F^0}\mathbf{F}_{i,0}, \quad \frac{d\phi_i^0}{dt} = \beta \frac{C}{d_0 F^0}\mathbf{M}_{i,0}. \tag{22}$$

Dimesionless parameters α and β thus set the external force-related bias in cell movements, while the noise in Eq. (20) gives rise to random walk-like movements.

Simulation Parameters

The unit distance of the simulations was set to the average size of an SPC111 cell adhering to a two dimensional substrate, $d_0 \approx$25 μm. The maximal distance, d_{max}, that still allows two cells to establish mechanical contact according to Eq. (4), was chosen as $d_{max} = 2d_0$. For elastic parameters we used values calibrated in [2]. SPC111 cells, like other metastatic cells, were assumed to be soft (E = 500 Pa, [8]). As cells can freely adjust their height within the contraction assay, the poisson number was assumed to be zero. These values translate to dimensionless microscopic parameters $k = 1.5$ and $g = 1$. We set the shear modulus associated with substrate adhesion to the same magnitude: $g_0 = 1.5$.

Our force unit – and the typical magnitude of the forces acting in the simulation – is the threshold force $F_0 \approx$ 100nN [2] which is needed to separate two adherent cells according to Eq. (3). Waiting times between simulation events are set by parameters A and B. We choose our time unit as $1/B \approx$ 10 min, the time needed for two adjacent cells to build up molecular complexes that mechanically link their cytoskeletons. We set the lifetime of an unloaded link to $1/A \approx$ 1 day. Thus, according to these values, two cells pulled away by the threshold force F_0 separate in $\approx$ 10 h, a value consistent with the time scale observed in our cell culture experiments. Similar time scales were set for contractility regulation ($1/C \approx$ 3 h), and for the force-induced cell drift ($1/\alpha \approx$ 2 h). According to the latter choice, the force unit F_0 induces a velocity drift of $\alpha d_0 \approx$ 12 μm/h – a value consistent with the typical cell speeds in our culture system.

Two dimensional initial conditions were generated by randomly positioning $N = 400$ particles in a square of size $L = 20$. The unit distance of the simulations was set to the average cell size, $d_0 \approx$10μm, thus the 2D cell density is 1 cell/unit area. In the initial condition we enforced that the distance of two adjacent particles is greater than $d_{min} = 0.8d_0$. Particles that are Voronoi neighbors are

connected by links when their distance is less than $d_{max} = 2d_0$. For a mechanical stress-free initial configuration we set the $\mathbf{t}_{i,l}$ preferred link direction vectors as well as the equilibrium link lengths ℓ_l so that no internal forces or torques are exerted in the system. As a boundary condition, we fixed the position and orientation of the particles located near the perimeter of the simulation domain.

Statistical analysis

A rectangular grid with uniform lattice spacing was layed over particle configurations, obtained either from simulations or optical-flow tracked experimental image sequences. For each grid cell we determined n, the number of particles located in that cell. Our measure of spatial inhomogeneity, S, is the standard deviation of the n values as

$$S = \sqrt{\overline{n^2} - \overline{n}^2}. \tag{23}$$

Two processes are compared using a point-by-point comparison of $S(t)$ values, obtained from parallel experiments or simulations. Statistical significance was established using a two-tailed t-test with p value below 5%.

The characteristic pattern size was determined using the power spectrum as

$$F^2(q) = \langle | \sum exp(-2\pi i \vec{q} \cdot \vec{x_j}) \; |^2 \rangle, \tag{24}$$

where each component of $\vec{q}$ is an integer multiple of $1/L$, and the average $\langle .. \rangle$ is calculated over vectors of the same length: $|\vec{q}| = q$. Power spectra were calculated for every 1000 steps of the stochastic simulations.

References

[1] G. I. Bell. Models for the specific adhesion of cells to cells. *Science*, 200(4342):618–627, May 1978.

[2] Andras Czirok and Dona Greta Isai. Cell resolved, multiparticle model of plastic tissue deformations and morphogenesis. *Phys Biol*, 12(1):016005, 2014.

[3] Daniel T. Gillespie. Exact stochastic simulation of coupled chemical reactions. *The Journal of Physical Chemistry*, 81(25):2340–2361, 1977.

[4] Eld Mhes, Beta Biri-Kovcs, Dona G Isai, Mrton Gulys, Lszl Nyitray, and Andrs Czirk. Matrigel patterning reflects multicellular contractility. *PLoS Comput. Biol.*, 15(10):e1007431, 10 2019.

[5] G. E. Rainger, C. D. Buckley, D. L. Simmons, and G. B. Nash. Neutrophils sense flow-generated stress and direct their migration through alphaVbeta3-integrin. *Am. J. Physiol.*, 276(3 Pt 2):H858–864, 1999.

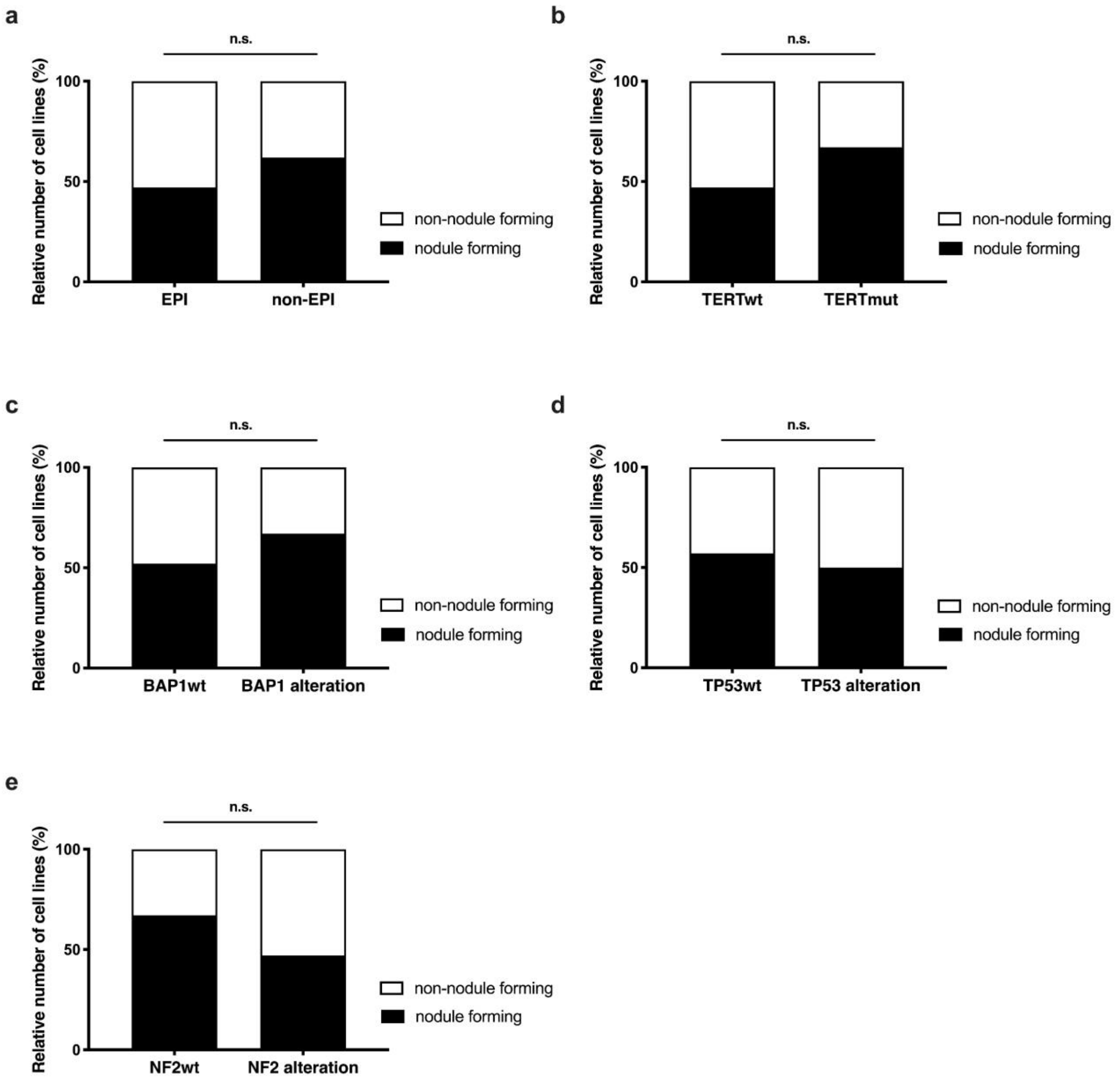

Supplementary Figure S1: Nodule formation does not associate with major MPM cell line - specific histological subtypes or mutational characteristics. MPM tumor cell lines were classified either as nodule forming or non-nodule forming. Bar charts display the prevalence of nodule forming cell lines within histological MPM subtypes (a), cell lines carrying wt or mutant TERT promoter (b), BAP1 (c), TP53 (d) or NF2 (e). The differences are not significant.

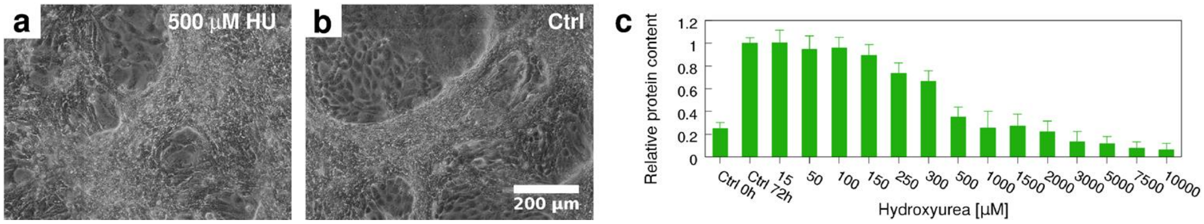

Supplementary Figure S2: Nodules appear even in the absence of cell proliferation. MPM cultures were incubated for 3 days in the presence (a) or absence (b) of 500 µM hydroxyurea (HU), an inhibitor of deoxyribonucleotide production, from the onset of nodule formation. Similar effect was seen in two independent experiments. The minimal effective hydroxyurea concentration was determined by measuring the total protein contents of HU treated and control cultures using the standard sulforhodamine B (SRB) assay (n=3, data are represented as means +/- standard deviation) (c).

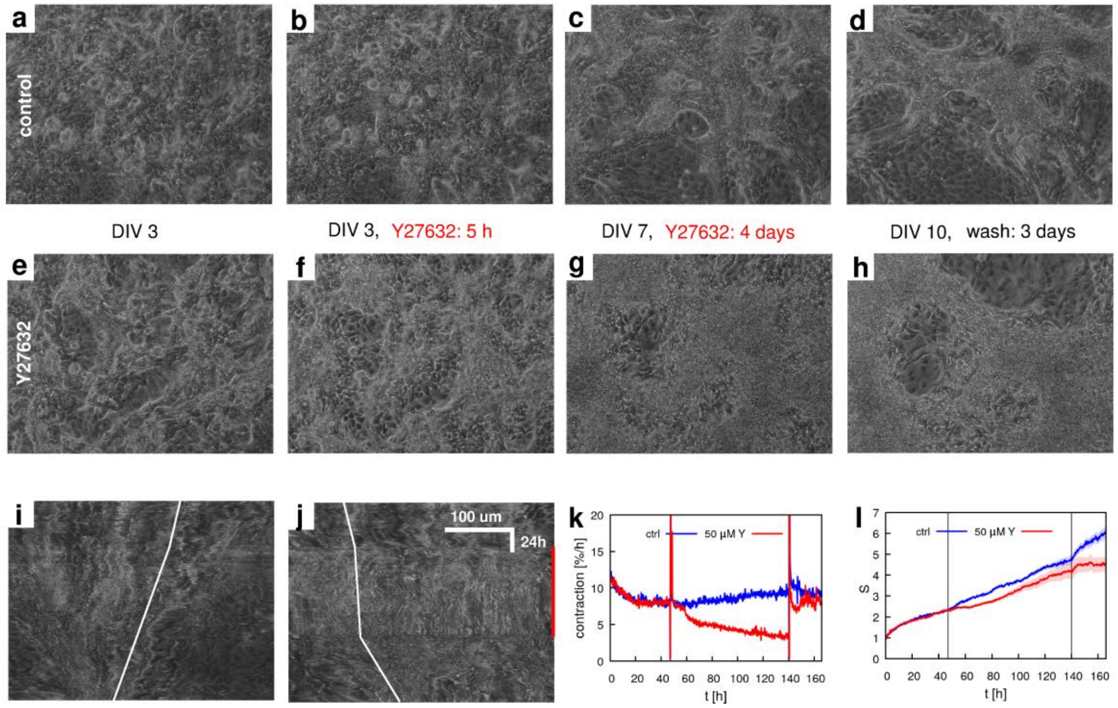

Supplementary Figure S3: Time course of in vitro nodule formation of SPC111 cell line, in the presence and absence of normal Myosin II activity. Frames from a time-lapse recording show the morphology of two parallel cultures: One was an untreated control (a-d) and the other was transiently exposed to 50 μM Y27632, a ROCK inhibitor (e-h). The two cultures are similar at 3 days in vitro (DIV), at the onset of treatment with Y27632 (a-e). A five hour-long exposure to Y27632 is sufficient to induce simultaneous flattening and lateral extension of the nodules (f). In untreated control cultures, dense three-dimensional cellular aggregates develop (c). Such aggregates are absent after 4 days of exposure to ROCK inhibitor (g). Removal of the drug restores the aggregation process of MPM cells: three days after replacing the medium, nodule morphologies are similar in both cultures (d-h). Kymogram (i-j) visualizes the aggregation process in control (i) and in Y27632 treated culture (j), where pixels along the same line are plotted for each frame. Lines from earlier frames are located at the top of the image, the total height of the image represents the 10 days of the experiment (i,j). Red line indicates the duration of Y27632 exposure (j). White lines are guides to the eye: the slope indicates contraction speed. See Supplementary Movie S1. Similar behavior was seen in five independent experiments. The progress of aggregation, evaluated by the contraction (k) and the standard deviation of tracing particle density, S (l), as a function of time t. The blue and red curves characterize untreated control cultures and cultures treated with 50 μM Y27632 ROCK inhibitor, respectively. The time interval when the culture was exposed to the inhibitor is delineated by vertical marker lines. Each curve is an average of n=4 recordings, the standard error of the mean is indicated by the shaded areas. The difference between the treated and untreated cultures is statistically significant at each time point after 100 h ($p<0.05$).

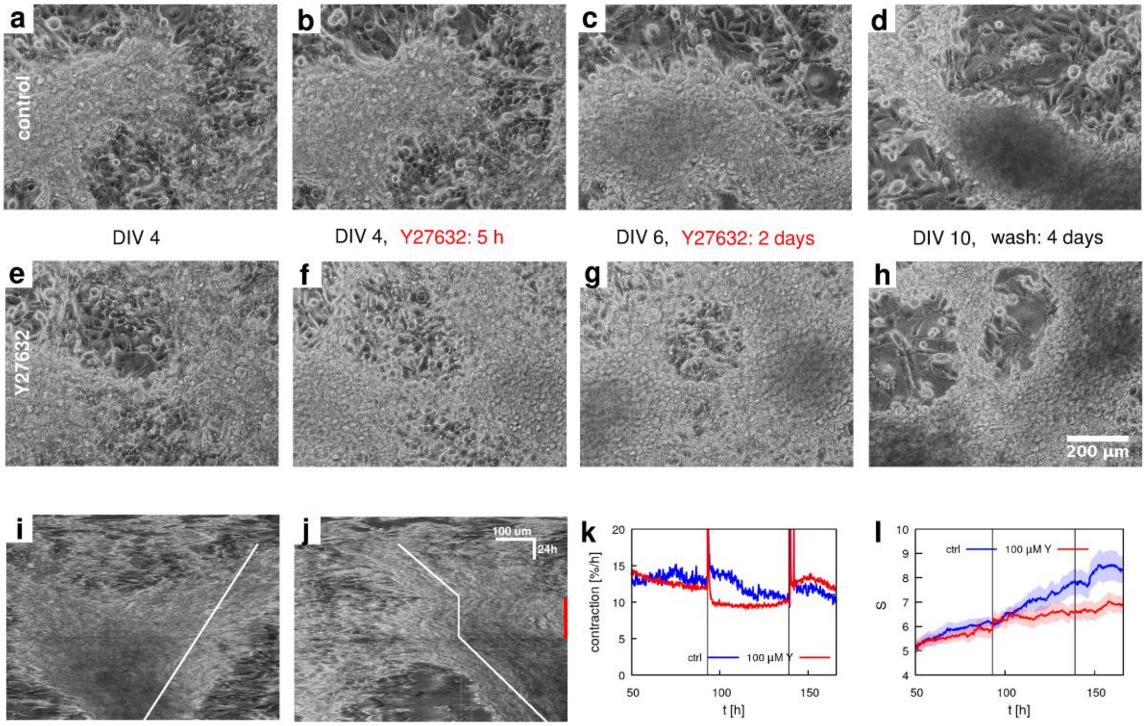

Supplementary Figure S4: Time course of in vitro nodule formation of VMC20 cell line, in the presence and absence of normal Myosin II activity. Frames from a time-lapse recording show the morphology of two parallel cultures: One was an untreated control (a-d) and the other was transiently exposed to 100 µM Y27632, a ROCK inhibitor (e-h). The two cultures are similar at 3 days in vitro (DIV), at the onset of treatment with Y27632 (a,e). A five hour-long exposure to Y27632 is sufficient to induce simultaneous flattening and lateral extension of the nodules (f). In untreated control cultures, dense three-dimensional cellular aggregates develop (c). Such aggregates are absent after 2 days of exposure to ROCK inhibitor (g). Removal of the drug restores the aggregation process of MPM cells: four days after replacing the medium, nodule morphologies are similar in both cultures (d,h). Kymogram (i,j) visualizes the aggregation process in control (i) and in Y27632 treated culture (j), where pixels along the same line are plotted for each frame. Lines from earlier frames are located at the top of the image, the total height of the image represents the 10 days of the experiment (i-j). Red line indicates the duration of Y27632 exposure (j). White lines are guides to the eye: the slope indicates contraction speed. Similar behavior was seen in two independent experiments. The progress of aggregation, evaluated by the contraction (k) and the standard deviation of tracing particle density, S, as a function of time t (l). The blue and red curves characterize untreated control cultures and cultures treated with 100 µM Y27632 ROCK inhibitor, respectively. The time interval when the culture was exposed to the inhibitor is delineated by vertical marker lines. Each curve is an average of n=8 recordings, the standard error of the mean is indicated by the shaded areas. . The difference between the treated and untreated cultures is statistically significant at each time point after 100h ($p<0.05$).

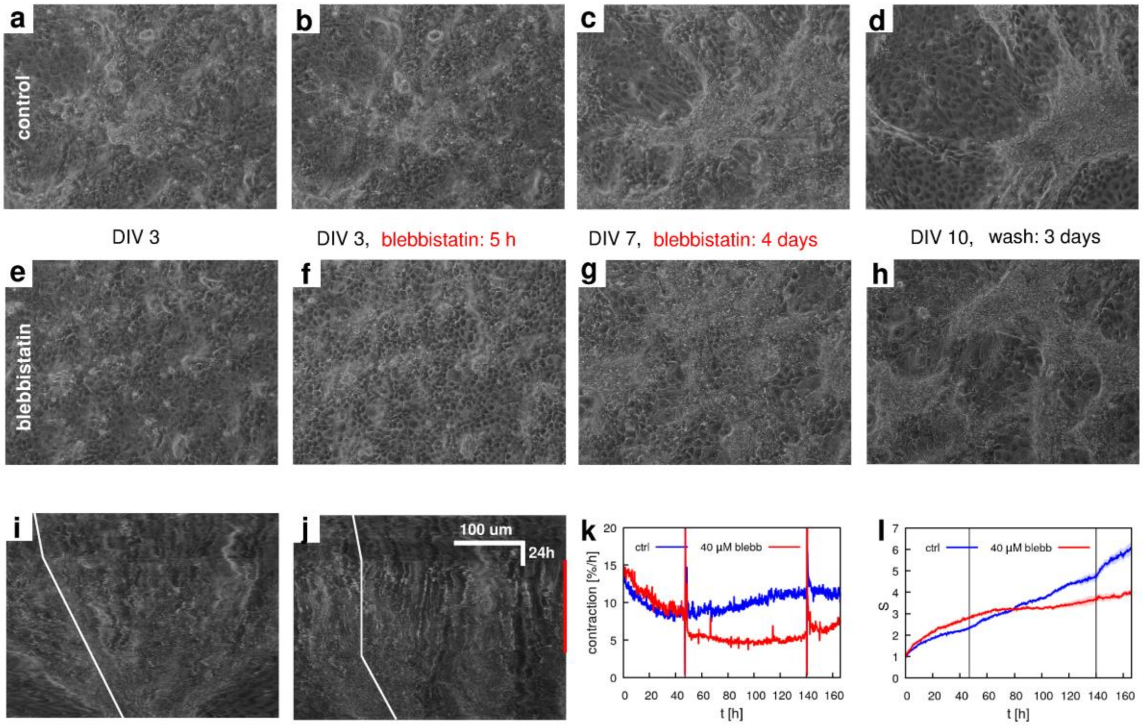

Supplementary Figure S5: Time course of in vitro nodule formation of SPC111 cell line, in the presence and absence of normal Myosin II activity. Frames from a time-lapse recording show the morphology of two parallel cultures: One was an untreated control (a-d) and the other was transiently exposed to 40 μM blebbistatin, a Myosin-II inhibitor (e-h). The two cultures are similar at 2 days in vitro (DIV), at the onset of treatment with blebbistatin (a,e). A five hour-long exposure to blebbistatin is sufficient to induce simultaneous flattening and lateral extension of the nodules (f). In untreated control cultures, dense three dimensional cellular aggregates develop (c). Such aggregates are absent after 4 days of exposure to Myosin-II inhibitor (g). Removal of the drug restores the aggregation process of MPM cells: three days after replacing the medium, nodule morphologies are similar in both cultures (d,h). Kymogram (i,j) visualizes the aggregation process in control (i) and in blebbistatin treated culture (j), where pixels along the same line are plotted for each frame. Lines from earlier frames are located at the top of the image, the total height of the image represents the 7 days of the experiment (i-j). Red line indicates the duration of blebbistatin exposure (j). White lines are guides to the eye: the slope indicates contraction speed. See Supplementary Movie S2. Similar behavior was seen in two independent experiments. The progress of aggregation, evaluated by the contraction (k) and the standard deviation of tracing particle density, S, as a function of time t (l). The blue and red curves characterize untreated control cultures and cultures treated with 40 μM blebbistatin Myosin-II inhibitor, respectively. The time interval when the culture was exposed to the inhibitor is delineated by vertical marker lines. Each curve is an average of n=4 recordings, the standard error of the mean is indicated by the shaded areas. The difference between the treated and untreated cultures is statistically significant at each time point after 100h ($p<0.05$).

MPM cell lines		non-MPM tumor cell lines	
**nodule forming**	**non-nodule forming**	**nodule forming**	**non-nodule forming**
I2	NP3 (normal pleura)	MEWO (melanoma)	A375 (melanoma)
M38K	CRL5820	DLD-1 (colorectal carcinoma)	M24met (melanoma)
Meso53	Meso49	A172 (glioblastoma)	VM15 (melanoma)
Meso62	Meso71	U87 (glioblastoma)	VM47 (melanoma)
Meso80	Meso84		HCA7 (colorectal carcinoma)
Meso92	Meso103		HCT116 (colorectal carcinoma)
Meso110	Meso189		HT29 (colorectal carcinoma)
Meso161	Meso194		SW1417 (colorectal carcinoma)
Meso204	Meso200		A549 (lung cancer)
Meso205	Meso221		CRL2066 (lung cancer)
Meso208	SPC212		CRL5922 (lung cancer)
P31cis	VMC6		H1650 (lung cancer)
SPC111	VMC12		HLHE (lung cancer)
VMC20	VMC23		GBM1 (glioblastoma)
VMC48	VMC40		T98 (glioblastoma)
			U373 (glioblastoma)

Supplementary Table S1. More than half of our MPM cell lines have capacity to form nodules, while normal primary mesothelial cells do not. Only one fifth of our non-MPM cancer cell lines are able to form nodules in vitro.

		Total (n=28)	nodule forming (n=15)	non-nodule forming (n=13)	*p*-value
**Histology**	epitheloid<br>non-epitheloid	15<br>13	7<br>8	8<br>5	0.476
**TERTp status**	TERTp^{wt}<br>TERTp^{mut}	19<br>9	9<br>6	10<br>3	0.435
**BAP1 (NA=1)**	BAP1 alteration*<br>BAP1^{wt}	6<br>21	4<br>11	2<br>10	0.662
**TP53 (NA=1)**	TP53 alteration*<br>TP53^{wt}	6<br>21	3<br>12	3<br>9	0.999
**NF2 (NA=1)**	NF2 alteration*<br>NF2^{wt}	15<br>12	7<br>8	8<br>4	0.441

*Loss of expression or mutation was considered alteration

Supplementary Table S2. Cell line specific characteristics dichotomized by nodule formation.

Supplementary Movie S1. Time-lapse phase-contrast video in vitro nodule formation in the presence or absence of normal Myosin II activity. After attachment of SPC111 cells, parallel cultures were either treated with 50 μM Y27632, a ROCK inhibitor for 4 days (96 h) or were left as untreated controls for comparison. After treatment the inhibitor was removed and imaging was continued for additional 6 days. During this period in the control culture dense 3D nodules develop, which process was inhibited by treatment with 50 μM Y27632.

Supplementary Movie S2. Time-lapse phase-contrast video of in vitro nodule formation in the presence or absence of normal Myosin II activity. After attachment of SPC111 cells, parallel cultures were either treated with 40 μM blebbistatin, a myosin II inhibitor for 4 days (96 h), or were left as untreated controls for comparison. After treatment the inhibitor was removed and imaging was continued for additional 6 days. In the presence of blebbistatin the emergence of contractile nodules was prevented, however 3D aggregates of rounded-up cells were present. Four days after removing the inhibitor contractile 3D nodules reappeared and were similar in both cultures. Abbreviation: 3D: three dimensional.

Supplementary Movie S3. Aggregation process of SPC111 cells in an untreated culture, monitored with virtual tracing particles. An SPC111 culture was imaged by phase-contrast time-lapse microscopy for 9 days. Displacements of virtual tracer particles, initially placed in a grid pattern, follow the optical flow and relocation of small image details. Higher tracer particle densities correspond to multicellular nodules.

Supplementary Movie S4. Aggregation process of SPC111 cells in the presence of ROCK inhibitor Y27632, imaged by phase-contrast time-lapse microscopy and monitored with virtual tracing particles. An SPC111 culture was treated with 100 μM Y27632 between days 3 and 7 (47-140 h), then the inhibitor was removed and culture was imaged for an additional 3 days. During the period of Y27632 treatment nodule formation is inhibited. Accordingly, the distribution of tracer particles remains more uniform. After the washout of the inhibitor nodules develop and the distribution of tracer particles becomes highly inhomogeneous.

Supplementary Movie S5. SPC111 nodules incorporate fluorescent bead-containing Matrigel substrate. SPC111 cells were seeded on fluorescent bead containing Matrigel and the same field was imaged by phase contrast (left) and epifluorescent (right) microscopy for 46 h. The experiment was repeated twice, yielding identical results.
